# Mitochondrial hyper-acetylation induced by an engineered acetyltransferase promotes cellular senescence

**DOI:** 10.1016/j.isci.2025.113233

**Published:** 2025-07-29

**Authors:** Tadahiro Shimazu, Ayane Kataoka, Takehiro Suzuki, Naoshi Dohmae, Yoichi Shinkai

**Affiliations:** 1 Cellular Memory Laboratory, RIKEN Cluster for Pioneering Research, Wako, Saitama 351-0198, Japan; 2 Biomolecular Characterization Unit, Technology Platform Division, RIKEN Center for Sustainable Resource Science, Wako, Saitama 351-0198, Japan

**Keywords:** Metabolic flux analysis, Metabolomics, Protein

## Abstract

Protein acetylation plays crucial roles in diverse biological functions, including mitochondrial metabolism. Although SIRT3 catalyzes the removal of acetyl groups in mitochondria, the addition of the acetyl groups is thought to be primarily controlled in an enzyme-independent manner due to the absence of potent acetyltransferases. In this study, we developed an engineered mitochondria-localized acetyltransferase, named engineered mitochondrial acetyltransferase (eMAT). eMAT localized in the mitochondrial matrix and introduced robust global protein lysine acetylation, including 413 proteins with 1,119 target lysine residues. Notably, 74% of the acetylated proteins overlapped with previously known acetylated proteins, indicating that the eMAT-mediated acetylation system is physiologically relevant. Functionally, eMAT negatively regulated mitochondrial energy metabolism, inhibited cell growth, and promoted cellular senescence, suggesting that mitochondrial hyper-acetylation drives metabolic inhibition and cellular senescence. SIRT3 counteracted eMAT-induced acetylation and metabolic inhibition, restored cell growth, and protected cells from senescence, highlighting the contribution of SIRT3 in maintaining energy metabolism and preventing cellular senescence.

## Highlights

• Engineered eMAT induces global lysine acetylation in mitochondria
• 74% of eMAT targets overlap with known mitochondrial acetyl-proteins
• eMAT-driven acetylation inhibits metabolism and promotes senescence
• SIRT3 reverses eMAT effects and protects against cellular senescence

## Graphical abstract

**Figure:**
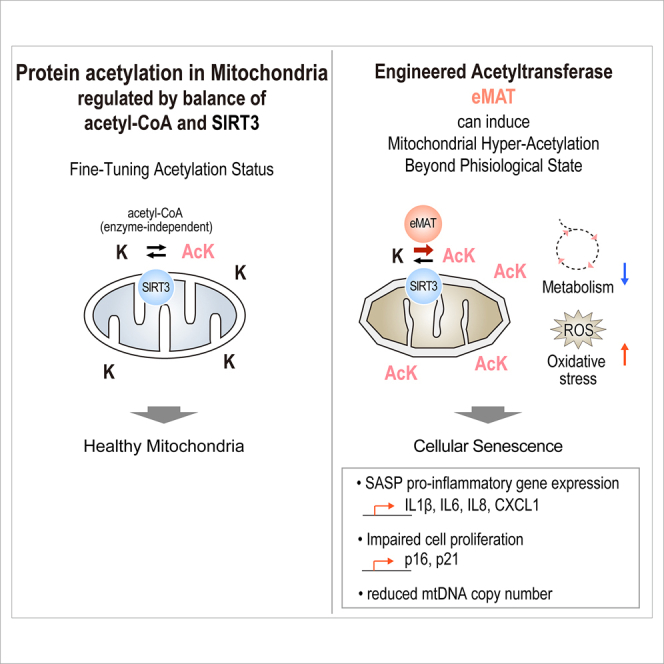

## Introduction

Lysine acetylation is a reversible post-translational modification (PTM) that occurs on more than 11,000 proteins in humans,^1^ which regulates gene expression, signal transduction pathways, metabolism, and other cellular processes.^2^^,^^3^ In particular, mitochondrial protein acetylation is prevalent among metabolism-related proteins, including enzymes involved in the TCA cycle and oxidative phosphorylation (OXPHOS). The acetylation of these proteins responds to nutritional alterations, such as a high-fat diet (HFD), fasting, and caloric restriction.^4^^,^^5^^,^^6^

Members of NAD^+^-dependent Class III HDACs, SIRT3, SIRT4, and SIRT5, are known to be localized in the mitochondria.^7^ Among them, SIRT3 is responsible for the deacetylation of mitochondrial proteins.^7^^,^^8^

Currently, no potent mitochondria-specific acetyltransferase has been characterized. Mitochondrial protein acetylation is predominantly thought to occur nonenzymatically, facilitated by the relatively high concentration of acetyl donors such as acetyl-CoA.^9^ Studies have shown that nonenzymatic acetylation can occur in a site-specific manner^9^^,^^10^ rather than through non-specific reactions, which may account for the selectivity and diversity of mitochondrial protein acetylation in the absence of enzymatic activity.

Given the lack of a potent mitochondrial acetyltransferase, altering SIRT3 activity has been the primary means to manipulate mitochondrial acetylation levels enzymatically. This limitation makes it challenging to evaluate lysine hyper-acetylation beyond *SIRT3* knockout models. In this study, we address this gap by introducing an engineered acetyltransferase molecule targeted to the mitochondria. This approach can manipulate mitochondrial lysine acetylation (AcK) and further clarify its regulatory roles in mitochondrial functions and cellular senescence.

## Results

### Design and characterization of engineered mitochondrial acetyltransferase

**Figure 1:**
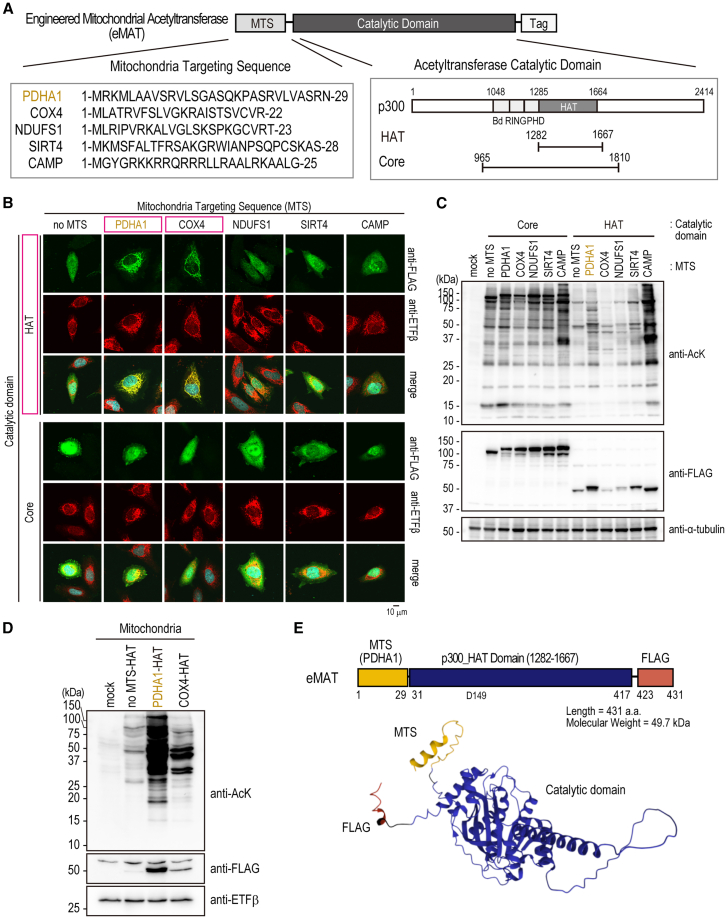
Design and characterization of engineered mitochondrial acetyltransferase; eMAT (A) Schematic structure of eMAT. Mitochondria targeting sequence (MTS) of PDHA1, COX4, NDUFS1, SIRT4, and CAMP were fused with p300 HAT domain (1282–1667 a.a.) or Core domain (965–1810 a.a.). (B) Subcellular localization of the fusion constructs (anti-FLAG, colored in green). PDHA1-HAT and COX4-HAT merged with a mitochondria marker protein ETFβ (colored in red). The blue signal indicates DAPI (nuclear marker). Box with magenta; constructs that showed mitochondrial localization. Scale bar: 10 μm. (C) Immunoblot images of total cell lysates from HEK293T transfected with indicated constructs. Top; anti-AcK antibody, Middle; anti-FLAG antibody, Bottom; anti-α-tubulin antibody as a loading control. (D) Immunoblot analysis of protein acetylation in mitochondria. Mitochondria were isolated from HEK293T cells transfected with indicated constructs, and immunoblot analysis was performed with anti-AcK (top), anti-FLAG (middle), and anti-ETFβ as mitochondria loading control (bottom). (E) Structure of eMAT. Top; schematic amino acid sequence of eMAT. Bottom; Structure of eMAT protein predicted with AlphaFold2^13^ based ColabFold program (v1.5.3).^14^

p300 is one of the most potent histone and non-histone protein lysine acetyltransferase. The core domain of p300 possesses acetyltransferase activity toward histones.^11^ Mitochondria proteins often have signal peptides with their N-terminus so-called mitochondria-targeting sequence (MTS). To obtain an artificial enzyme that is located in the mitochondria, we constructed a series of chimeric molecules of p300 catalytic domains fused with N-terminal mitochondria-targeting sequences (Figure 1A).

After our initial screening, HAT domain (1282–1667 a.a. from human p300) or Core domain (965–1810 a.a.) was fused with N-terminal MTS from PDHA1 (1–29 a.a.), COX4 (1–22 a.a.), NDUFS1 (1–23 a.a.), SIRT4 (1–28 a.a.), and a synthetic peptide CAMP (1–25 a.a.)^12^ with C-terminal FLAG tag sequence. Their localization was observed with a fluorescent microscope (Figure 1B). Within tested, PDHA1_MTS-HAT and COX4_MTS-HAT were specifically localized in the mitochondria. All fusion acetyltransferases could enhance AcK with similar but distinct substrate specificities (Figure 1C). Among the two, PDHA1_MTS-HAT could introduce more robust AcK in the mitochondria compared to COX4_MTS-HAT (Figure 1D). From these results, we decided to further characterize the PDHA1_MTS-HAT fusion protein as a mitochondrial acetyltransferase and therefore renamed it as engineered Mitochondrial Acetyltransferase (eMAT). The structure of eMAT was predicted in silico with the AlphaFold2 program,^13^^,^^14^ which demonstrates a modular structure of N-terminal MTS sequence derived from PDHA1, central p300 catalytic domain, and C-terminal FLAG tag sequence (Figure 1E).

### eMAT specifically catalyzes lysine acetylation in mitochondria and negatively regulates cell proliferation

**Figure 2:**
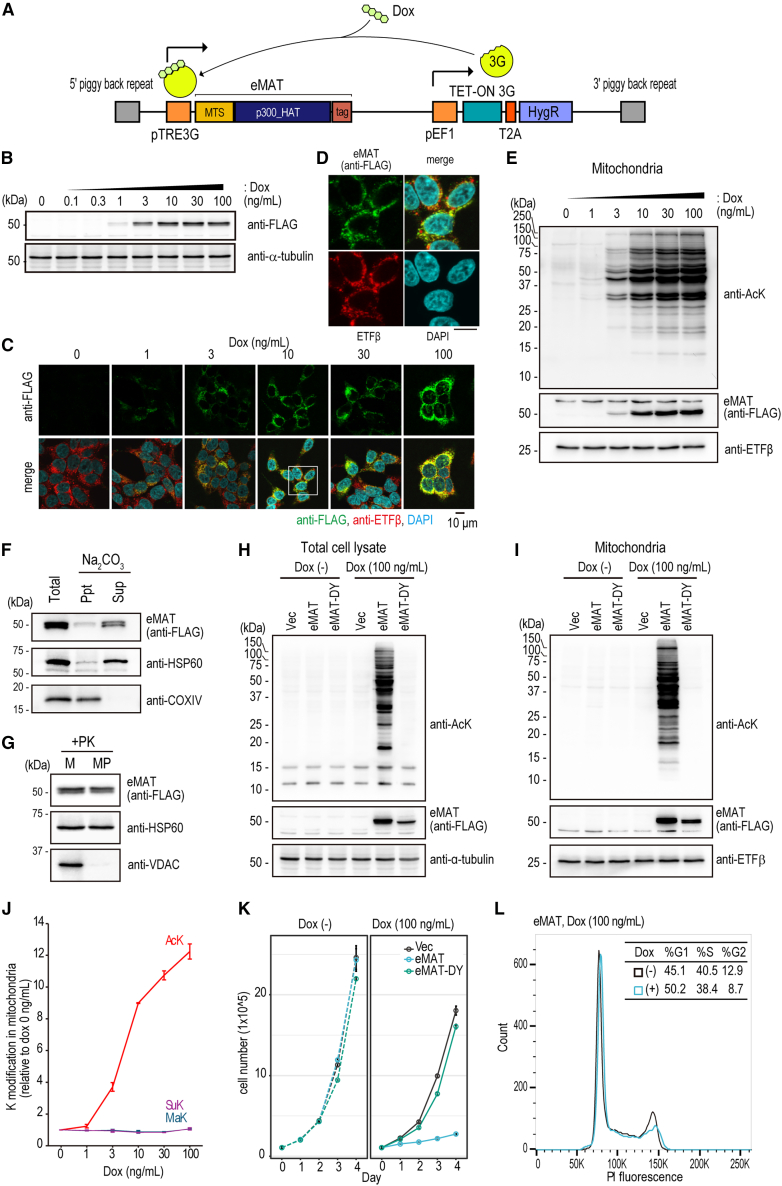
eMAT specifically catalyzes lysine acetylation in mitochondria and negatively regulates cell proliferation (A) A schematic for Dox inducible eMAT expression system. (B) Dox-dependent expression of eMAT. 0 to 100 ng/mL of Dox were treated for 24 h, and the expression of eMAT was determined by immunoblotting of total cell lysates with anti-FLAG antibody (top) and anti-α-tubulin antibody (bottom). See also Figures S1A–S1E for K modifications. (C) Immunofluorescent microscopic analysis of eMAT. Scale bar: 10 μm. The square box indicates the enlarged area in (D). (E) Typical immunoblot image of Dox-dependent AcK in the mitochondria. K succinylation (SuK) and K malonylation (MaK) in mitochondria fractions were detected as well (Figures S1F and S1G). (F) Submitochondrial fractionation of eMAT. Total mitochondria (total) were treated with sodium carbonate buffer (Na2CO3) followed by ultra-centrifugation to obtain membrane fraction as pellets (ppt) and remaining soluble supernatant (Sup). Proteins in each fraction were analyzed by immunoblot. HSP60; a marker for soluble fraction, COXIV; a marker for membrane fraction. (G) Total mitochondria (M) were treated with or without hypotonic buffer to obtain mitoplast (MP). Followed by a Proteinase K treatment, proteins in MP or M were analyzed. HSP60; a marker for the matrix, VDAC; a marker for the outer membrane. Empty vector transfected control (Vec), eMAT, or its catalytic mutant (eMAT-DY) inducible cells were treated with or without Dox for 24 h, and their acetylation in total cell lysate (H) or mitochondria fraction (I) was determined by immunoblot with anti-AcK antibody. Immunoblots with anti-SuK and anti-MaK were shown in Figures S1F and S1G. (J) K acylations in eMAT cells. AcK, SuK, and MaK in mitochondria fractions were determined with corresponding modification-specific antibodies (Figures 2E, S1H, and S1I), and their signal intensities were quantified with ImageJ software. mean ± SEM; *n* = 2. (K) control (vec), eMAT or catalytic mutant (eMAT-DY) inducible cells (1x10ˆ5) were cultured in 37°C 5% CO2 incubator in the presence or absence of Dox (100 ng/mL), and their cell numbers were counted at indicated time point. mean ± SEM; *n* = 3. (L) Cell cycle distribution of eMAT cells treated with or without Dox (100 ng/mL) for 48 h. See also Figure S2.

To investigate acetylation introduced by eMAT, we established doxycycline (Dox)-inducible eMAT-expressing HEK293T cells using a piggyBac-Dox-inducible overexpression system (PB-TRE,^15^; Figure 2A). The expression level of eMAT was tightly regulated by the concentration of Dox in the culture medium (Figure 2B), and its mitochondrial localization was confirmed by immunofluorescence microscopy at concentrations up to 100 ng/mL of Dox (Figures 2C and 2D). Furthermore, we observed dose-dependent mitochondrial acetylation following Dox treatment (Figures 2E, S1A, and S1B).

To determine submitochondria localization of eMAT, the isolated mitochondria from eMAT-expressing cells were treated with an alkali buffer (100 mM Na_2_CO_3_, pH 11.5), and ultracentrifuged to separate insoluble fraction (ppt) and soluble fraction (sup). eMAT was fractionated in the sup, suggesting its non-membrane localization (Figure 2F). In parallel, the isolated mitochondria were treated with a hypotonic buffer (20 mM HEPES-KOH, pH 7.5) to obtain mitoplast (MP), and the mitoplast or mitochondria (M) were treated with 100 ng/mL proteinase K to remove surface-bound proteins. eMAT was present in the PK-treated mitoplast (Figure 2G). From these results, we conclude that eMAT is localized in the mitochondrial matrix.

eMAT expression induced robust mitochondrial acetylation (Figures 2H and 2I). To confirm that the acetylation depends on eMAT activity, a catalytic mutant of eMAT (eMAT-DY), in which D149 (corresponding to enzymatically essential D1399 of p300^11^) was substituted with Y, was constructed. eMAT-DY failed to induce acetylation, confirming that the catalytic activity of eMAT is essential for mitochondrial acetylation. To further characterize the enzymatic activity of eMAT, lysine malonylation (MaK) and lysine succinylation (SuK) in total cell lysates (Figures S1C–S1E) and mitochondrial fraction (Figures 2J and S1F–S1J) were examined by immunoblot analysis with pan modification-specific antibodies. eMAT did not induce MaK nor SuK up to 100 ng/mL Dox treatment, whereas it induced AcK as low as 1 ng/mL Dox, demonstrating that eMAT predominantly functions as a lysine acetyltransferase (Figure 2J).

We next tested if eMAT induces histone acetylation, although no obvious nuclear localization was observed under the microscopic analysis (Figures 2C and 2D). Acetylation of histone H3 was apparently increased in cells transfected with p300 Core domain as well as full-length p300 (Figure S2A), whereas eMAT expressing cells induced almost no H3 acetylation up to 10 ng/mL Dox, and showed a slight increase (1.5-fold) when treated with >30 ng/mL Dox (Figures S2A and S2B), confirming eMAT primary function as a mitochondrial acetyltransferase (Figure S2C).

To date, GCN5L1^16^^,^^17^ and KAT8/MOF^18^^,^^19^ have been identified as modulators of mitochondrial acetylation. Mitochondrial acetylation induced by eMAT was compared with that induced by GCN5L1 or KAT8. We found that eMAT-mediated mitochondrial acetylation was much more robust than that induced by GCN5L1 or KAT8 under our experimental conditions, demonstrating that eMAT is a potent engineered enzyme for mitochondrial acetylation (Figures S2D and S2E). To compare mitochondrial hyper-acetylation between eMAT and *SIRT3* knockout (KO), CRISPR-Cas9 mediated *SIRT3* KO cells were established (Figures S2F–S2H). *SIRT3* KO modestly (1.5-fold) enhanced global mitochondrial acetylation, whereas eMAT could drastically induce them (>10-fold) (Figures S2I and S2J). These observations indicate eMAT is a potent tool to induce mitochondrial hyper-acetylation beyond the physiological level.

Since mitochondrial acetylation links to energy metabolism,^2^^,^^3^ we next tested whether eMAT affects cell growth rate. Proliferation of Dox-inducible eMAT cells was monitored for 5 days in the presence or absence of Dox (100 ng/mL) (Figure 2K). The proliferation rate was decreased in wild-type eMAT-expressing cells but not in eMAT-DY cells, suggesting eMAT-mediated cell growth inhibition was acetyltransferase activity-dependent. Flow cytometric analysis revealed that eMAT cells had less G2 population and more G1 population in the presence of Dox for 48 h (Figure 2L). We observed no increase in dead cell population by eMAT induction (Figures S2K–S2N). These indicate that eMAT had a negative effect on cell cycle progression.

### SIRT3 counteracts eMAT-induced mitochondrial acetylation and restores cell proliferation

**Figure 3:**
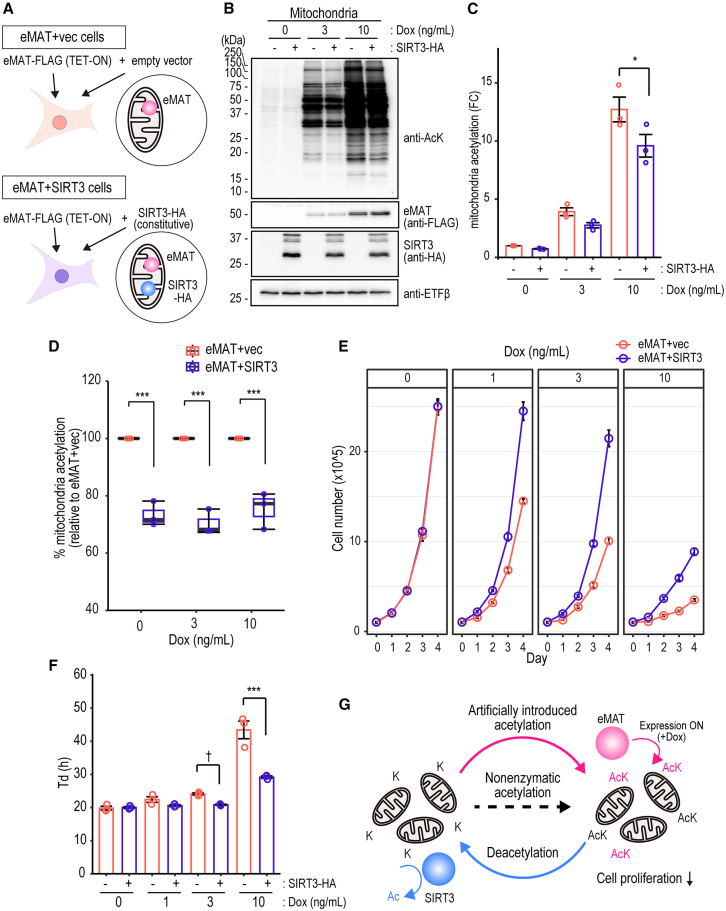
SIRT3 counteracts eMAT-induced mitochondrial acetylation and restores cell proliferation (A) A Schematic for Dox-inducible eMAT cells with empty vector (eMAT+vec) or SIRT3-HA (eMAT+SIRT3). See also Figure S3. (B) eMAT+vec or eMAT+SIRT3 cells were treated with the indicated amount of Dox for 24 h. Representative immunoblot image of mitochondrial acetylation determined with anti-AcK antibodies. (C) Quantitation of mitochondrial acetylation. Relative mitochondrial acetylation of eMAT (light blue) or eMAT+SIRT3 cells (pink) treated with 0–10 ng/mL of Dox was determined according to the intensity of the anti-AcK blot after normalization with the intensity of anti-ETFβ as loading control using ImageJ software. *n* = 3; mean ± SEM. Tukey’s HSD test: *p*∗ = 0.033. (D) Relative quantitation of mitochondrial acetylation. *n* = 3; mean ± SEM. Tukey’s HSD test: *p*∗∗∗ < 0.001. (E) 1x10ˆ5 cells were treated with or without Dox (0–10 ng/mL), cultured in 37°C 5% CO_2_ incubator, and their cell numbers were counted for 5 days *n* = 3; mean ± SEM. (F) Doubling time (Td) of eMAT or eMAT+SIRT3 in the presence or absence of Dox. *n* = 3; mean ± SEM. FDR-adjusted pairwise T-test: *p*† = 0.069, *p*∗∗∗ < 0.001. (G) A schematic of reversible enzymatic acetylation by eMAT and SIRT3.

SIRT3 is a Class III deacetylase localized in the mitochondria and is responsible for mitochondrial deacetylation.^7^ We hypothesized that SIRT3 could erase the eMAT-introduced acetylation mark. To evaluate this, the Dox-inducible eMAT cells were infected with retroviruses containing either an empty vector (eMAT+vec) or a vector encoding SIRT3 with a C-terminal HA tag (eMAT+SIRT3), resulting in Dox-inducible eMAT cells with SIRT3-HA overexpression (Figures 3A and S3A–S3C). Mitochondrial acetylation level in each cell line was assessed in the absence of Dox (no eMAT expression) or the presence of 3 ng/mL Dox (low eMAT expression) or 10 ng/mL Dox (high eMAT expression) (Figures 3B and 3C). SIRT3 overexpression reduced ∼30% of total mitochondrial acetylation in these conditions, suggesting SIRT3 deacetylates eMAT-induced acetylation besides endogenously occurring acetylation (Figure 3D).

Next, we addressed whether eMAT-mediated cell proliferation inhibition was recovered by SIRT3. eMAT+vec cells or eMAT+SIRT3 cells were treated with Dox (0–10 ng/mL), and their cell proliferations were monitored for 5 days (Figure 3E). Doubling time (Td) of eMAT+vec cells vs. eMAT+SIRT3 cells was unchanged without Dox treatment (20 h), whereas 24.1 h vs. 20.9 h (*p*-value = 0.069) under low eMAT expression (3 ng/mL Dox) and 43.4 h vs. 29.2 h (*p*-value < 0.001) under high eMAT expression (10 ng/mL Dox), implying that SIRT3 overexpression restored the cell proliferation inhibition by eMAT (Figure 3F). These results indicate that the eMAT-introduced mitochondrial acetylation negatively regulates cell proliferation, and SIRT3 could erase these acetylation marks and restore cell proliferation (Figure 3G).

### Global mitochondrial acetylation controlled by eMAT and SIRT3

**Figure 4:**
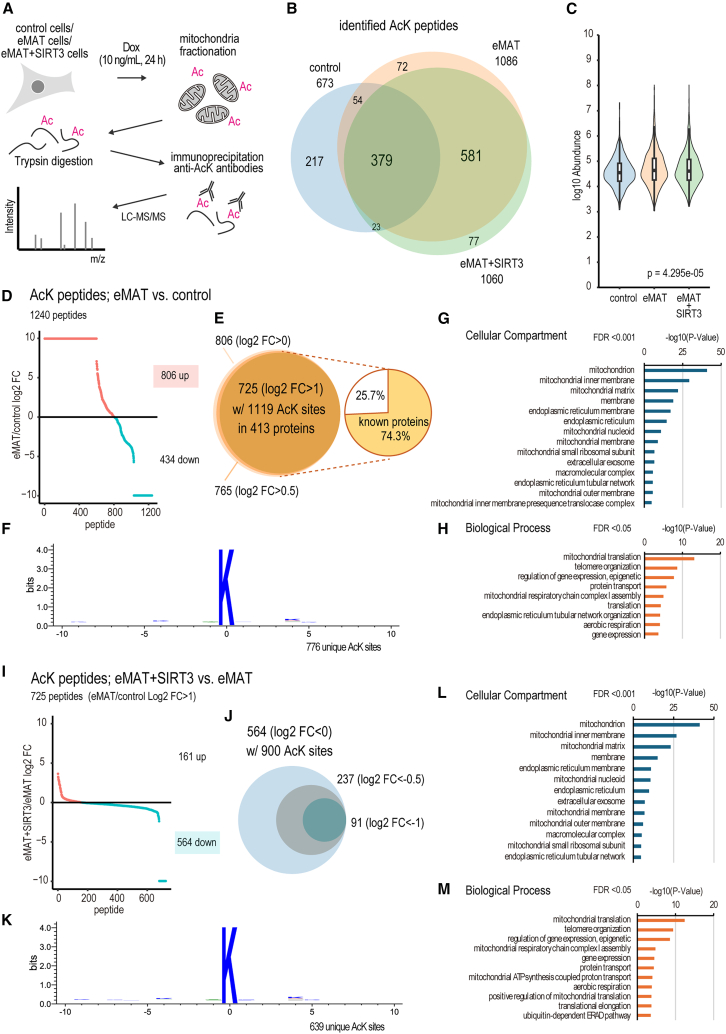
Global mitochondrial acetylation controlled by eMAT and SIRT3 (A) A schematic for quantitative LC-MS/MS. Mitochondria fractions from parental, eMAT treated with 100 ng/mL Dox, eMAT+SIRT3 treated with 100 ng/mL Dox were isolated and treated with trypsin. The tryptic peptides were immunoprecipitated with anti-AcK antibodies, and captured with Protein-A/G agarose beads. The beads-bound peptides were eluted and analyzed with LC-MS/MS with a label-free quantitative method (Proteome Discoverer Ver.3.1). (B) Venn diagram of identified AcK-containing peptides. (C) Violin plot of the AcK peptides. Friedman Test: *p* = 4.295 × 10^−5^. (D) Comparison of acetylation between eMAT and control sample. Fold change of acetylated peptides (total 1240 peptides) was calculated as log2([peptide abundance of eMAT]/[peptide abundance of control]). (E) Ven diagram of the eMAT targets. Among 725 identified substrates with log2FC > 1, 74.3% were known proteins. (F) Consensus motif analysis of acetylation sites with WebLogo (Ver.3). GO analysis of eMAT targets with DAVID (Ver.2021): (G) Cellular compartment, (H) Biological process. (I) Comparison of acetylation between eMAT+SIRT3 and eMAT. Fold changes were calculated as log2([peptide abundance of eMAT+SIRT3]/[peptide abundance of eMAT]). (J) Ven diagram of the eMAT and SIRT3 targets. (K) Consensus motif analysis of deacetylation sites by SIRT3. GO analysis of eMAT and SIRT3 targets: (L) Cellular compartment, (M) Biological process.

To identify proteins acetylated by eMAT, acetylome analysis was performed using anti-acetylated lysine-specific antibodies. Briefly, parental HEK293T cells (control), eMAT cells, and eMAT+SIRT3 cells were treated with 10 ng/mL of Dox for 24 h to induce eMAT expression and mitochondrial acetylation (Figure 4A). Their Mitochondria were isolated, and the mitochondrial fractions were digested with trypsin. The tryptic peptides were immunoprecipitated with a cocktail of anti-AcK antibodies, and precipitated peptides were analyzed with LC-MS/MS with a label-free quantitation method (Tables S1 and S2). A total of 1403 peptides were identified in this study. The control sample showed fewer peptides (673) as compared to those in eMAT (1086) or eMAT+SIRT3 (1060) (Figure 4B). 68% of identified peptides (456) in the control cells overlapped with eMAT or eMAT+SIRT3, suggesting eMAT targets native acetylation sites that were detected in the control. There is a remarkable (88%) overlap between the identified peptides in eMAT and eMAT+SIRT3, which implies that SIRT3 can target proteins acetylated by eMAT. The overall peptide abundance was different between the three conditions, which may reflect a variety of their protein acetylation levels (Figure 4C).

To better understand the eMAT-catalyzed acetylation, the abundance of identified peptides in the control sample and eMAT sample was compared, and we found 806 peptides were enriched (log2 fold change (log2FC) > 0) in eMAT cells (Figure 4D). Among the 806 peptides, 765 peptides had log2FC > 0.5, and 725 peptides with 1119 AcK sites in 413 proteins showed log2FC > 1 (Figure 4E; Tables S1 and S2). Here, we defined the 725 peptides with log2FC > 1 as acetylated peptides by eMAT. 74.3% of the identified targets were known acetylated proteins according to a public database (PhosphoSitePlus), suggesting eMAT-mediated acetylation widely overlapped with native acetylation. A consensus motif analysis of eMAT substrates was carried out with 776 unique acetylation sites by eMAT, demonstrating almost no preference around the target lysine sites (Figure 4F). This implies that eMAT is a global mitochondrial lysine acetyltransferase with no clear sequence specificity. Gene ontology (GO) analysis confirmed that the majority of acetylated proteins by eMAT were mitochondria-associated proteins (Figures 4G and 4H).

Next, peptides found in eMAT and eMAT+SIRT3 were compared. Among the 725 eMAT target peptides, 564 peptides with 900 AcK sites were reduced in SIRT3 over-expression (log2FC < 0), whereas only 91 peptides showed more than 2-fold reduction (log2FC < −1, Figures 4I and 4J). The modest decrease in eMAT+SIRT3 could be explained by the observation that acetylation and deacetylation were dynamic and equilibrium reactions between SIRT3 and eMAT (Figure 3). Here, we defined the 564 peptides with log2FC < 0 as SIRT3 targets. A consensus motif analysis of the SIRT3 targets with 639 unique deacetylation sites was performed, which again showed no preference around target lysine sites (Figure 4K). This indicates that SIRT3 deacetylates eMAT targets with no sequence preference, confirming that SIRT3 functions as a major mitochondrial deacetylase. GO analysis revealed that the eMAT- and SIRT3-double substrates are localized in mitochondria and involved in diverse mitochondrial functions such as mitochondrial translation or energy metabolism (Figures 4L and 4M).

### eMAT acetylates multiple mitochondrial metabolic enzymes and SOD2

**Figure 5:**
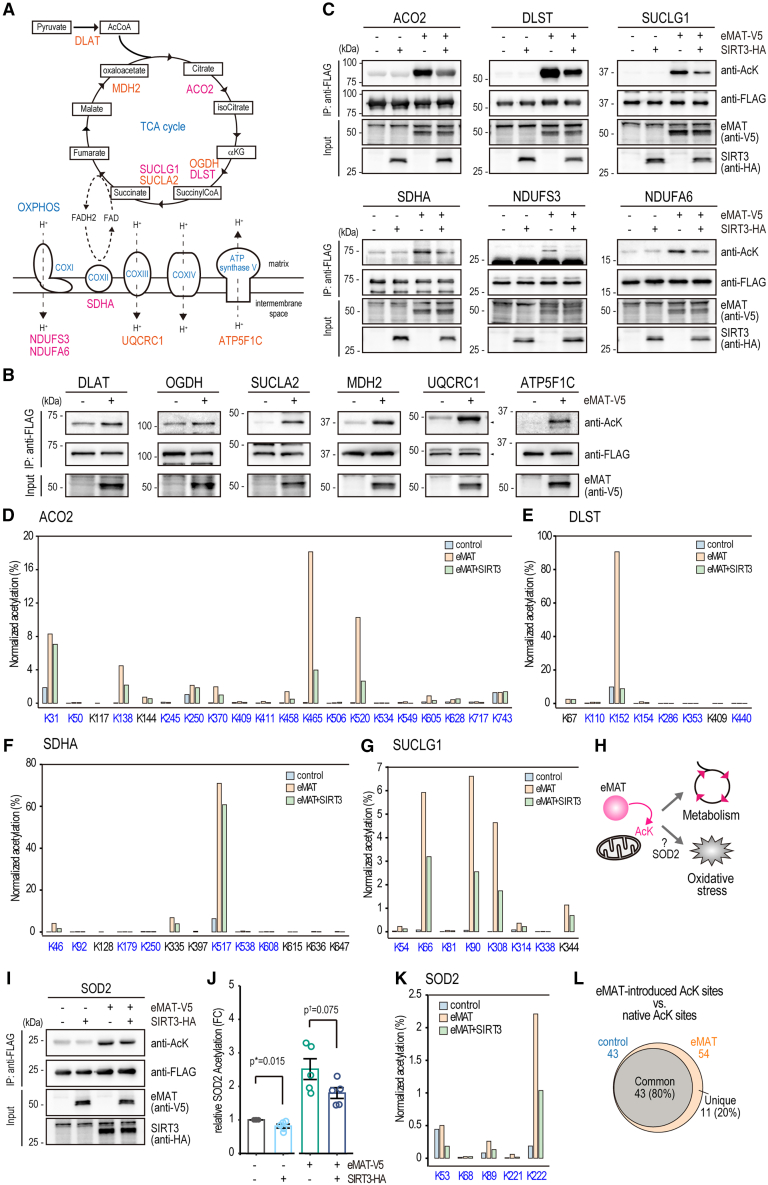
eMAT acetylates multiple mitochondrial metabolic enzymes and SOD2 (A) Validated metabolic enzymes acetylated by eMAT: Orange; eMAT-dependent acetylation, magenta; eMAT-dependent acetylation and SIRT3-dependent deacetylation. FLAG-tagged proteins were expressed in Dox-inducible eMAT cells (B) or eMAT+Vec and eMAT+SIRT3 cells (C), treated with 10 ng/mL Dox for 24 h. Immunoprecipitated proteins were blotted with anti-AcK antibodies or anti-FLAG antibodies. Expression of eMAT (anti-V5) and SIRT3 (anti-HA) was confirmed with immunoblot of total cell lysates. LC-MS/MS analysis of FLAG-tagged (D) ACO2, (E) DLST, (F) SDHA, (G) SUCLG1 in control (eMAT-V5+vec cells without Dox treatment), eMAT (eMAT-V5+vec cells treated with 10 ng/mL Dox for 24 h), eMAT+SIRT3 (eMAT-V5+SIRT3 treated with 10 ng/mL Dox for 24 h) cells. Normalized acetylation (%) was calculated based on the intensity of the acetylated peptide, normalized by the sum of the intensities of the corresponding unacetylated and acetylated peptides. Blue; common acetylation site whose acetylation was detected both in the control and eMAT. (H) Schematic of eMAT mediated acetylation of metabolic enzymes and SOD2. (I) Representative immunoblot of eMAT-dependent acetylation of SOD2. (J) Quantitation of SOD2 acetylation. *n* = 5; mean ± SEM. Tukey’s HSD test: *p*† < 0.1, *p*∗ < 0.05. (K) LC-MS/MS analysis of FLAG-tagged SOD2 in control, eMAT, eMAT+SIRT3 cells. (L) Venn diagram showing the number of acetylation sites identified in control and eMAT conditions across the five substrates.

The acetylome analyses (Figures 4 and S4; Tables S1, S2, and S3) revealed that multiple metabolic enzymes are potential substrates for eMAT-mediated acetylation (Figure S4G). To validate these acetylations, we picked up twelve metabolic enzymes (Figure 5A), cloned them with a c-terminal FLAG tag, and transfected them into Dox-inducible V5-tagged eMAT cells (Figure S3). Their eMAT-dependent acetylation was detected with immunoblot analysis (Figures 5B and 5C). Subsequently, SIRT3-dependent deacetylation of aconitase 2 (ACO2), dihydrolipoamide S-succinyltransferase (DLST), succinate-CoA ligase GDP/ADP-forming subunit alpha (SUCLG1), succinate dehydrogenase complex flavoprotein subunit A (SDHA), NADH:ubiquinone oxidoreductase core subunit S3 (NDUFS3) and NADH:ubiquinone oxidoreductase subunit A6 (NDUFA6) was confirmed (Figure 5C). LC-MS/MS identified multiple acetylation sites on ACO2 (Figure 5D), DLST (Figure 5E), SDHA (Figure 5F), and SUCLG1 (Figure 5G), with their acetylation levels controlled by eMAT and SIRT3.

SOD2 acetylation level and its enzymatic activity are controlled by SIRT3.^20^^,^^21^^,^^22^ Since eMAT and SIRT3 largely overlap in their substrates, eMAT may target SOD2 as well (Figure 5H). To test this, eMAT-dependent SOD2 acetylation was detected with the immunoblot analysis (Figure 5I). The acetylation was increased with eMAT expression. Consistent with previous reports that SIRT3 deacetylates SOD2,^20^^,^^21^^,^^22^ the acetylation was decreased with SIRT3 overexpression (Figure 5J). LC-MS/MS identified multiple acetylation sites in SOD2, which were controlled by eMAT and SIRT3 (Figure 5K). These results demonstrate that eMAT and SIRT3 coordinately regulate the acetylation of multiple metabolic enzymes and the reactive oxygen species (ROS) scavenger, SOD2.

Among the 54 acetylation sites identified in the eMAT-expressing cells, 43 sites (80%) overlapped with those identified in the control (Figures 5D–5G, and 5K, colored with blue), suggesting that eMAT preferably introduces acetylation in native sites (Figure 5L). This result is consistent with the observation that the majority of eMAT-substrates were known acetylated proteins (Figure 4E). Taken together, eMAT mainly targets lysines that can be acetylated under native conditions while also possessing the ability to generate additional unique acetylation sites.

### eMAT regulates energy metabolism

**Figure 6:**
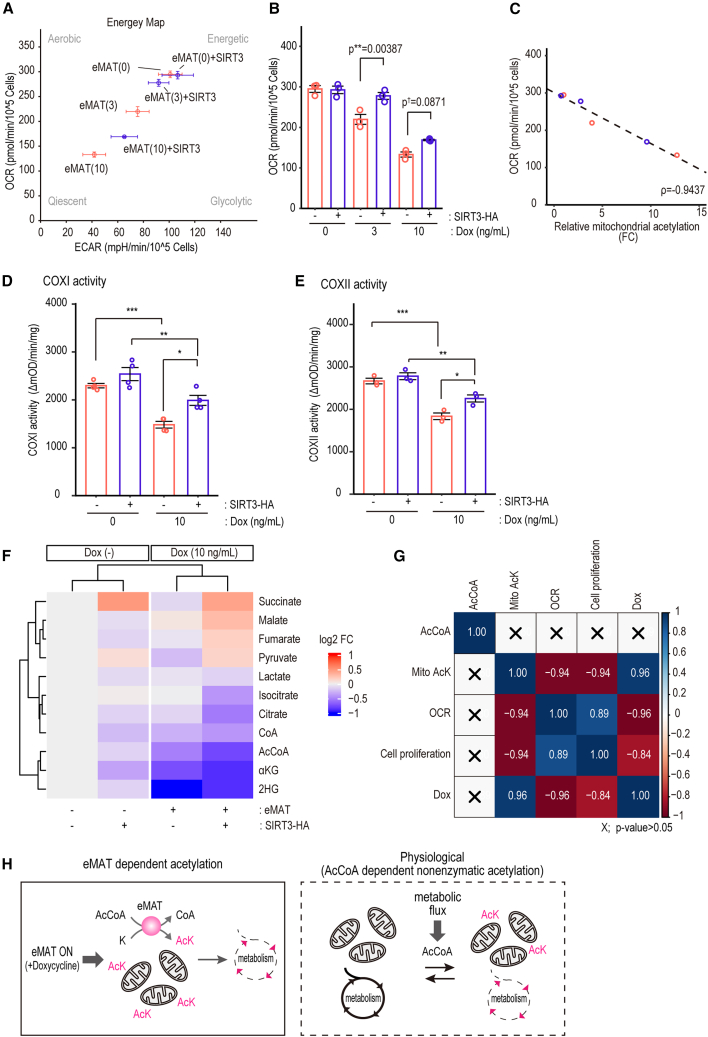
eMAT regulates energy metabolism (A) Extracellular flux analysis. ECAR and OCR were measured with XFe96(Agilent). (B) OCR was calculated from the first 3 baselines of the mitostress test (see also Figure S5A). *n* = 3; mean ± SEM. Tukey’s HSD test: *p*† < 0.1, *p*∗∗ < 0.01. (C) Correlation of mitochondrial acetylation and OCR. OCR and relative mitochondrial acetylation in eMAT+vec and eMAT+SIRT3 (Figure 3C) were plotted. Spearman’s rank correlation coefficient: ρ = −0.9437. *In vitro* (D) COXI and (E) COXII activity assay were performed with isolated mitochondria. *n* ≥ 3; mean ± SEM. Tukey’s HSD test: *p*∗ < 0.05, *p*∗∗ < 0.01, *p*∗∗∗ < 0.001. (F) Heatmap of metabolome analysis. eMAT+vec or eMAT+SIRT3 cells were treated with or without 10 ng/mL Dox for 24 h. The concentration of each metabolite was obtained as pmol/L×10ˆ6 cells (see also Figure S5B), and log2FC to control cells (eMAT+vec cells without Dox treatment) was calculated and presented. Three biological replicates were analyzed. (G) Spearman correlation plot of acetyl-CoA (AcCoA), mitochondrial lysine acetylation (mito AcK), OCR, cell proliferation, and Dox concentration. Correlations with *p*-values less than 0.05 were shown. (H) Schematic comparison of eMAT-dependent acetylation and physiological acetylation.

We examined whether eMAT regulates energy production since eMAT-mediated acetylation links to energy metabolism (Figures 4H and 4M) and eMAT acetylates multiple metabolic enzymes (Figure 5). Dox-inducible eMAT and eMAT+SIRT3 cells were treated with 0, 3, and 10 ng/mL of Dox for 24 h, and extracellular acidification rate (ECAR) and oxygen consumption rate (OCR) were monitored. As expected, eMAT expression repressed both ECAR and OCR, and co-expression of SIRT3 partially restored them (Figures 6A, 6B, and S5A). The mitochondrial acetylation level inversely correlated with OCR, suggesting mitochondrial acetylation induced by eMAT negatively regulates mitochondrial OXPHOS (Figure 6C). To further investigate the OXPHOS activities in eMAT, *in vitro* activity assays were performed. We found that eMAT inhibited Complex I (COXI, Figure 6D) and Complex II (COXII, Figure 6E) activities, and SIRT3 partially recovered them.

Next, a metabolome analysis of TCA cycle intermediates was performed (Figures 6F and S5B). Induction of eMAT downregulated almost all TCA metabolites, whereas SIRT3 altered the balance of intracellular TCA metabolites (i.e., accumulation of succinate and reduction of α-ketoglutarate). Coexpression of eMAT and SIRT3 augments the metabolic shifts seen in the eMAT expression. The decrease of TCA intermediates in eMAT supports the observation that eMAT suppresses OXPHOS activity (Figures 6A–6E).

Consumption of acetyl-CoA by eMAT itself may impact OXPHOS activity since enzymatic acetylation utilizes acetyl-CoA as an acetyl-donor. To evaluate this, a correlation analysis of intra-cellular acetyl-CoA level with mitochondria acetylation, OCR, cell growth rate, and Dox concentration was performed (Figure 6G). Mitochondrial acetylation correlated with Dox concentration and inversely correlated with OCR or cell proliferation. On the other hand, acetyl-CoA concentration showed no statistically significant correlation (*p*-value > 0.05) with any of them, suggesting that protein acetylation, rather than acetyl-CoA concentration, plays a crucial role in the regulation of OXPHOS and cell proliferation in the eMAT-expressing cells (Figure 6H left).

eMAT-expressing cells showed decreased acetyl-CoA levels (Figures 6F and S5B) with higher mitochondrial acetylation as compared to control cells. This indicates that eMAT can efficiently introduce acetylation even under less acetyl-CoA concentration due to its high affinity to acetyl-CoA (Figure S6A). The Km value for acetyl-CoA of p300, the catalytic module for eMAT, is 1.2 μM,^23^^,^^24^ which is far less required for nonenzymatic acetylation.^9^^,^^10^ Several studies measured cellular acetyl-CoA to be 10–30 μM and estimated mitochondrial acetyl-CoA concentrations to be around several mM.^9^^,^^10^^,^^25^^,^^26^^,^^27^ To determine acetyl-CoA concentrations in eMAT cells, we measured acetyl-CoA concentration in the total cell lysate (Figure S6B). We could not precisely determine mitochondrial acetyl-CoA because of the instability of the metabolite during mitochondrial fractionation. Based on the previous estimation of yeast mitochondrial acetyl-CoA concentration,^27^ we estimate mitochondrial acetyl-CoA to be 30-fold higher than whole cell, which results in around 0.8–1 mM in eMAT-expressing cells, a similar range as previous reports.^9^^,^^10^^,^^25^^,^^26^ To compare eMAT-mediated enzymatic acetylation with nonenzymatic acetylation, recombinant ACO2 and NDUFS3 proteins were reacted with varying concentrations of acetyl-CoA in the presence or absence of eMAT, and their acetylation was determined *in vitro* (Figures S6C–S6F). Acetylation of ACO2 and NDUFS3 occurred as low as 10 μM in the presence of eMAT, whereas nonenzymatic acetylation occurs around a mM range, confirming that eMAT can catalyze mitochondrial acetylation at fur less acetyl-CoA concentration, thereby inducing hyper-acetylation in living cells.

Nonenzymatic acetylation is dependent on acetyl-CoA concentration,^27^ and local acetyl-CoA levels in the nucleus have been shown to influence histone acetylation.^28^ Similarly, an increase in mitochondrial acetyl-CoA levels may promote the acetylation of metabolic enzymes, which in turn can regulate both acetyl-CoA production and consumption. This feedback mechanism may contribute to the fine-tuning of mitochondrial metabolism in response to environmental or nutritional conditions (Figure 6H, right).

### eMAT promotes senescence in hTERT-RPE1 cells

**Figure 7:**
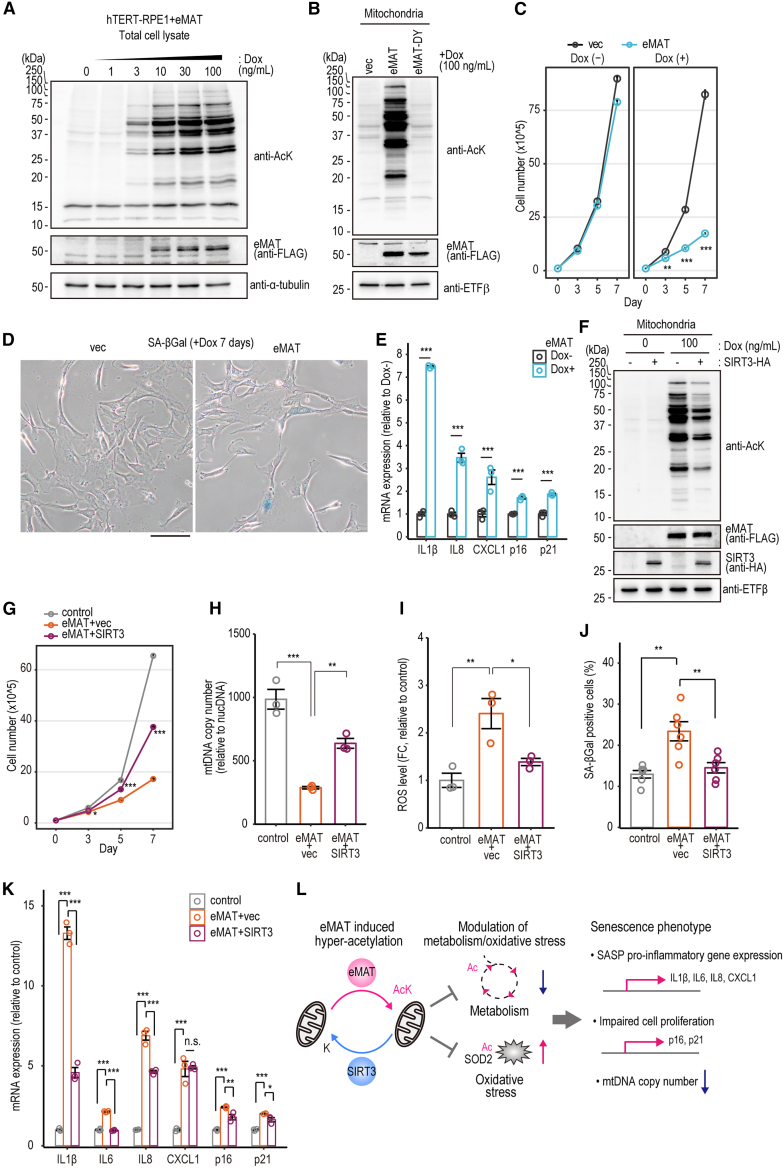
eMAT promotes senescence in hTERT-RPE1 cells (A) Representative immunoblots of Dox-dependent AcK in Dox-inducible eMAT cells in hTERT-RPE1. (B) Representative immunoblots of mitochondrial acetylation in Dox-inducible eMAT cells. (C) 1x10ˆ5 hTERT-RPE1 cells stably transfected with control vector or eMAT were cultured in the absence or presence of Dox (100 ng/mL) and their cell numbers were counted at indicated periods. mean ± SEM; *n* = 3. Student’s *t* test: *p*∗∗ < 0.01, *p*∗∗∗ < 0.001. (D) Control (vec) or eMAT-inducible (eMAT) hTERT-RPE1 cells were cultured in the presence of Dox (100 ng/mL) for 7 days, then stained with X-gal at pH 6.0 to detect SA-βGal positive cells. A representative bright-field image was shown. Scale bar: 100 μm (E) mRNA expression of SASP-related pro-inflammatory gene in the eMAT-inducible hTERT-RPE1 cells with or without Dox (100 ng/mL, 7 days). mean ± SEM; *n* = 3. Student’s *t* test: *p*∗∗∗ < 0.001. (F) A representative immunoblot image of eMAT- and SIRT3-dependent mitochondrial acetylation in hTERT-RPE1 cells. See also Figure S3. (G) Cell numbers of control (eMAT+vec without Dox), eMAT (eMAT+vec with 100 ng/mL Dox), or eMAT+SIRT3 (eMAT+SIRT3 with 100 ng/mL Dox) were counted at indicated time point (start from 1x10ˆ5 cells at Day 0). mean ± SEM; *n* = 3. Tukey’s HSD test: *p*∗ < 0.05, *p*∗∗∗ < 0.001. (H) Control (PB-TRE-vector), eMAT (eMAT+vec) and eMAT+SIRT3 hTERT-RPE1 cells were cultured with 100 ng/mL Dox for 7 days mtDNA copy number was determined with a real-time qPCR with primers specific to mtDNA (mitochondrial tRNALeu^(UUR)^) and nucDNA (nuclear β2M). mean ± SEM; *n* = 3. Tukey’s HSD test: *p*∗∗ < 0.01, *p*∗∗∗ < 0.001. (I) Mitochondrial ROS were detected with a mitochondrial superoxide indicator (MitoSOX Green). Each Cell line was treated with 100 ng/mL Dox for 7 days before the staining. The fluorescent images were quantified with ImageJ software. mean ± SEM; *n* = 3. Tukey’s HSD test: *p*∗ < 0.05, *p*∗∗ < 0.01. See also Figure S7A. (J) Control, eMAT, or eMAT+SIRT3 hTERT-RPE1 cells were stained with X-gal at pH 6.0, and the SA-βgal positive cells were counted. mean ± SEM; *n* = 6. Tukey’s HSD test: *p*∗∗ < 0.01. (K) mRNA expression of SASP-related pro-inflammatory gene in control, eMAT or eMAT+SIRT3 hTERT-RPE1 cells. mean ± SEM; *n* = 3. Tukey’s HSD test: *p*∗ < 0.05, *p*∗∗ < 0.01, *p*∗∗∗ < 0.001. (L) A schematic model of mitochondrial hyper-acetylation and cellular senescence.

We examined whether eMAT-induced mitochondrial acetylation contributes to cellular senescence, given that eMAT expression caused G1 cell-cycle arrest and inhibited proliferation (Figure 2), acetylated multiple metabolic enzymes and SOD2 (Figure 5), and suppressed mitochondrial OXPHOS (Figure 6). To assess senescence, normal human diploid hTERT-RPE1 cells were transfected with a Dox-inducible eMAT plasmid and selected with G418 (800 μg/mL) for two weeks to generate stable cell populations (Figure S3A). Dox-dependent eMAT expression (Figure 7A) and eMAT activity–dependent mitochondrial hyperacetylation were confirmed (Figure 7B). Cell proliferation was inhibited in eMAT-expressing cells in the presence of 100 ng/mL Dox (Figure 7C). To assess senescence-associated β-galactosidase (SA-βGal) activity,^29^ eMAT-expressing hTERT-RPE1 cells were stained with X-Gal at pH 6.0. An increased proportion of SA-βGal–positive cells was observed, indicating that eMAT promotes cellular senescence (Figure 7D). Furthermore, RT-qPCR analysis revealed upregulation of senescence-associated secretory phenotype (SASP) markers (IL1β, IL8, CXCL1) and cell cycle regulators (p16, p21)^30^ in eMAT-expressing hTERT-RPE1 cells (Figure 7E).

To investigate whether SIRT3 confers protection against senescence in eMAT-expressing hTERT-RPE1 cells, retroviral vectors encoding SIRT3-HA were introduced into eMAT-expressing cells, followed by selection with blasticidin (10 μg/mL) for two weeks to establish stable cell populations (Figure S3D). Increased mitochondrial protein acetylation was observed in eMAT-expressing cells, and this acetylation was partially suppressed in cells co-expressing eMAT and SIRT3 (Figure 7F). Furthermore, the impaired cell growth observed in eMAT-expressing cells was partially rescued in cells co-expressing SIRT3 (Figure 7G).

To further assess senescence-associated mitochondrial phenotypes, we evaluated mitochondrial DNA (mtDNA) copy number, mitochondrial ROS levels, and mitochondrial mass following treatment with Dox (100 ng/mL) for 7 days. The mtDNA copy number was decreased in eMAT-expressing cells, and this reduction was partially restored by co-expression of SIRT3 (Figure 7H). Mitochondrial ROS levels were elevated in eMAT-expressing cells, but not in control or eMAT+SIRT3-expressing cells (Figures 7I and S7A). Mitochondrial mass remained unchanged across these cell lines (Figure S7B). eMAT expression led to an increase in SA-βGal–positive cells, whereas co-expression of SIRT3 mitigated this effect (Figure 7J). Finally, RT-qPCR analysis revealed elevated expression of SASP markers (IL1β, IL6, IL8) and cell cycle regulators (p16, p21) in eMAT-expressing cells (Figure 7K), which was reversed by SIRT3 co-expression, indicating a protective role of SIRT3 in eMAT-induced cellular senescence (Figure 7L).

## Discussion

Protein lysine acetylation regulates diverse cellular functions, including gene expression, signal transduction, and energy metabolism.^2^ In humans, over 20 types of HATs have been identified and categorized into various families based on their structural domains.^31^^,^^32^ Prominent families include the P300/CBP family (e.g., P300 and CBP), the GNAT family (e.g., GCN5 and PCAF), and the MYST family (e.g., MOZ, Tip60, MOF, and HBO1). Furthermore, certain transcription factors, such as TFIIIC (a universal transcription factor for RNA polymerase III) and CLOCK (an epigenetic regulator of circadian rhythms), are also recognized as HATs. Among them, GCN5L1^16^^,^^17^ and KAT8/MOF^18^^,^^19^ have been identified as mitochondria-associated acetyltransferases, although their contribution to the mitochondrial acetylation is not fully uncovered.

To further elucidate the physiological significance of mitochondrial protein AcK, we designed an artificial mitochondrial acetyltransferase, which we named eMAT (Figure 1). eMAT specifically localized in the mitochondrial matrix (Figure 2). We confirmed that the expression of eMAT leads to a global hyperacetylated state of mitochondrial proteins. As compared to known mitochondria-associated acetyltransferases GCN5L1 and KAT8, eMAT introduced robust mitochondrial acetylation (Figure S2). Enhanced acetylation of mitochondrial proteins has been observed in cases such as SIRT3 knockdown (approximately 2.5-fold increase) or mitochondria derived from mice fed an HFD (approximately 1.5-fold increase).^6^ In this report, *SIRT3* KO HEK293T cells showed about a 1.5-fold increase in global mitochondrial acetylation (Figure S2J). The acetylation level induced by eMAT was over 10-fold in the mitochondria (Figure 4), which is remarkably higher than any previous reports to our best knowledge.

eMAT catalyzes the acetylation of diverse substrates without a clear sequence specificity surrounding the target lysine residues. The majority of acetylation by eMAT occurred in native sites, suggesting eMAT-catalyzed acetylation is physiologically relevant (Figures 4E and 5L). In fact, eMAT affected multiple biological processes such as OXPHOS, cell growth, and cellular senescence. Although we examined the physiological role of eMAT in the context of energy metabolism and cellular senescence in this report, eMAT-induced acetylation may also impact other mitochondrial functions such as mitochondrial translation or gene expression as revealed by acetylome analyses (Figures 4H and 4M). Additional Studies are required to clarify these biological impacts.

The broad reactivity of eMAT has the potential to induce off-target effects. To address this, we evaluated both the subcellular localization of eMAT and its impact on histone acetylation. eMAT predominantly localized to mitochondria at Dox concentrations up to 100 ng/mL (Figure 2C), with only a modest increase (approximately 1.5-fold) in histone H3 acetylation (Figures S2A–S2C). While we cannot completely rule out the possibility that increased histone acetylation or other non-mitochondrial acetylation may contribute to the physiological effects observed in this study, it is noteworthy that SIRT3 was able to counteract the phenotypes induced by eMAT. In the next step, it may be possible to further minimize off-target effects by incorporating a degron sequence into eMAT, given that mitochondria lack the ubiquitin-proteasome system. In addition, the broad reactivity of eMAT makes it challenging to modulate acetylation of specific substrates, although our aim in this report is to manipulate global mitochondrial acetylation levels. To achieve substrate-specific acetylation, it may be possible to employ or engineer an alternative HAT domain beyond that in p300. Additionally, tethering the optimal protein interaction motifs could help guide the biomolecules to their desired targets.

SIRT3, an NAD^+^-dependent class III HDAC, is located in the mitochondrial matrix and deacetylates diverse substrates.^7^ SIRT3 can remove the artificially introduced acetylation by eMAT and thereby alleviate the negative impact on cell growth and energy metabolism. Many studies have reported that SIRT3 inactivation leads to hyper-acetylation which results in metabolic defects. For instance, it has been reported that SIRT3 regulates complex I activity^33^ and complex II activity through deacetylation of NDUFA9^34^ and SDHA.^35^ In this study, we reciprocally demonstrated that acetylation by eMAT resulted in both COXI and COX II inhibition (Figure 6), confirming that induction of acetylation, in addition to inactivation of the deacetylase SIRT3, is crucial for the regulation of energy metabolism.

Cellular senescence is a state in which cells permanently stop dividing but remain metabolically active. It serves as a protective mechanism against cancer by preventing the uncontrolled proliferation of damaged cells. However, the accumulation of senescent cells is also associated with aging and age-related diseases. In this report, we found that eMAT promotes cellular senescence in normal human diploid h-TERT RPE1 cells (Figure 7). eMAT may trigger stress-induced premature senescence (SIPS) through perturbation of energy metabolism and elevation of oxidative stress since eMAT acetylates multiple mitochondrial metabolic enzymes and the antioxidant enzyme SOD2 whose acetylation is linked to suppression of their enzymatic activities.^20^^,^^21^^,^^22^ Mitochondrial dysfunction, as illustrated by impaired mitochondrial biogenesis and increased production of ROS, leads to age-related neurodegenerative diseases such as Alzheimer’s and Parkinson’s disease.^36^^,^^37^ Sirtuins are deacetylases that have been shown to regulate both the replicative and overall lifespan in model organisms, including C. elegans and Drosophila melanogaster.^38^^,^^39^ Here we revealed that a mitochondria-localized sirtuin, SIRT3, ameliorated eMAT-induced metabolic defects and prevented eMAT-expressing hTERT-RPE1 cells from senescence.

HFD conditions may represent a physiological state in which acetyl-CoA concentrations are elevated due to enhanced fatty acid oxidation (FAO).^40^ Mitochondrial hyperacetylation has been reported under HFD conditions,^6^ and HFD has also been shown to increase cellular senescence in glial cells in a mouse model,^41^ suggesting a potential link between mitochondrial hyperacetylation and cellular senescence during HFD. Additionally, a recent study demonstrated that enhanced FAO, driven by the medium-chain fatty acid octanoate, induces cellular senescence via increased histone acetylation, which promotes p16 expression through the nuclear acetyl-CoA pool.^42^ It is plausible that FAO stimulation by octanoate also elevates mitochondrial protein acetylation, which could also contribute to cellular senescence via modulating metabolism and ROS production. Furthermore, a previous study reported that mitochondrial acetyl-CoA metabolism is altered during oncogene-induced senescence (OIS) through PDH activity.^43^ Given that PDH activity is regulated by both phosphorylation and acetylation, and that we observed increased acetylation of the E2 subunit of PDH (DLAT, Figure 5), it is possible that its physiological acetylation contributes to cellular senescence. Although we demonstrated that artificially induced mitochondrial hyper-acetylation by eMAT promotes cellular senescence by modulating metabolism largely independent of acetyl-CoA levels (Figure 6), it remains important to determine to what extent mitochondrial hyperacetylation under physiological conditions, such as HFD or FAO activation, contributes to the induction of cellular senescence. This is particularly relevant given that acetyl-CoA serves as a substrate for both mitochondrial protein acetylation and nuclear histone acetylation, thereby linking metabolism to gene regulation. In addition, the relationship between eMAT-induced mitochondrial hyper-acetylation and neurodegenerative diseases or lifespan in mammals should be examined.

It has been proposed that sirtuins and their substrates are evolutionarily related since cytoplasmic/nuclear sirtuin (SIRT1) and mitochondrial sirtuin (SIRT3) target their homologous substrates.^44^ This raises the question of whether cytoplasmic/nuclear acetyltransferases and mitochondrial acetyltransferases similarly evolved in parallel with the divergence of deacetylase families. Given the unfavorable consequence of mitochondrial hyper-acetylation by eMAT, cells/organisms may be evolutionally selected not to have a potent acetyltransferase that continuously drives a global mitochondria hyper-acetylation. On the other hand, organisms acquired the mitochondrial deacetylase, SIRT3, to protect cells from acetylation-induced metabolic and oxidative stresses that could trigger cellular senescence.

In this study, we conducted manipulation of mitochondrial protein acetylation by an engineered acetyltransferase, eMAT, which could regulate metabolic status, cellular proliferation, and cellular senescence. Significant efforts have been made to manipulate gene expression using engineered biomolecules, such as dCas9 fused with histone acetyltransferases, deacetylases, or methyltransferases.^45^ The enzyme-dCas9 complex introduces or removes modifications at specific positions on histones to regulate gene expression. These advancements have drawn attention to epigenetics research employing artificial PTMs. Also, the ubiquitin-proteasome system is widely used to manipulate protein levels in living cells/organisms. PROTAC system^46^^,^^47^^,^^48^ or auxin-inducible degron (AID) system^49^ introduces polyubiquitination on designated proteins to control their protein levels at desired situations. However, to our knowledge, no attempts have been made to introduce artificial acetylation via engineered enzymes in non-histone molecules to manipulate their physiological functions. As demonstrated in this study, manipulation of PTMs is an attractive strategy for controlling physiological functions in cells even outside of the nucleus, as these modifications are rapid, reversible, and inherently sophisticated, given that organisms have been naturally equipped with these mechanisms over long periods. Our methodology provides a new system for inducing mitochondrial acetylation at a desired timing other than knockout/knockdown of the deacetylase SIRT3, offering a valuable approach for analyzing the mitochondrial protein hyper-acetylation which promotes cellular senescence. Additional studies are required to clarify whether the fluctuation of mitochondrial acetylation under a certain physiological state or nutrient environment also links to cellular senescence and aging.

### Limitations of the study

While eMAT is designed to target mitochondria, it may induce off-target effects. We observed a modest increase in histone acetylation upon eMAT expression, suggesting that nuclear or other non-mitochondrial acetylation could also contribute to the physiological effects observed in this study. Additionally, we did not delineate the extent to which physiological mitochondrial acetylation is functionally involved in the regulation of cellular senescence. Finally, as this study was conducted exclusively in a cell line model, it remains unclear whether eMAT induces cellular senescence or aging in animal models or humans.

## Resource availability

### Lead contact

Requests for further information and resources should be directed to and will be fulfilled by the lead contact, Tadahiro Shimazu (tshimazu@riken.jp).

### Materials availability

• This study did not generate new unique reagents.
• Plasmids and/or cell lines generated in this study are available upon reasonable request and completion of a material transfer agreement (MTA). Please contact the lead contact for further information.

### Data and code availability

• Source data for the main figures are provided in the manuscript or in the Supplemental Information. The mass spectrometry proteomics data have been deposited in the ProteomeXchange Consortium under the dataset identifiers PXD061245 and PXD061246, as listed in the key resources table.
• This paper does not report any original code.
• Any additional information required to reanalyze the data reported in this paper is available from the lead contact upon request.

## Acknowledgments

We are grateful to Dr. Akihiro Ito for providing human p300 and SIRT3 cDNA constructs. We thank the staff of the Research Resources Division, RIKEN Center for Brain Science, for their support with DNA sequencing, flow cytometry, and LC-MS/MS. We especially thank Masaya Usui for assistance with MRM-based quantification. This work was supported by the 10.13039/501100001691Japan Society for the Promotion of Science Grant-in-Aid for Transformative Research Areas (B) [22H05020 to T.S.].

## Author contributions

T. Shimazu designed and performed experiments, analyzed the data, and prepared the manuscript; A.K. performed experiments; T. Suzuki and N.D. performed and analyzed mass spectrometry; Y.S. supervised the study, planned experiments, interpreted data, and prepared the manuscript.

## Declaration of interests

The authors declare no competing interests.

## STAR★Methods

### Key resources table

**Table:** 

REAGENT or RESOURCE	SOURCE	IDENTIFIER
**Antibodies**		

Anti-Succinyllysine rabbit pAb	PTM BIO	Cat#PTM-401; RRID:AB_2687628
Malonyl-Lysine [Mal-K] multiMab Rabbit mAB mix	Cell Signaling Technology	Cat#14942; RRID:AB_2687627
Acetylated Lysine (Ac-K2-100) MultiMab Rabbit mAb mix	Cell Signaling Technology	Cat#9814; RRID:AB_10544700
Acetylated-Lysine Antibody	Cell Signaling Technology	Cat#9441; RRID:AB_331805
SIRT3 (D22A3) Rabbit mAb	Cell Signaling Technology	Cat#5490; RRID:AB_10828246
COXIV mouse mAb	Cell Signaling Technology	Cat#11967; clone#4D11-B3-E8; RRID:AB_2797784
VDAC (D73D12) Rabbit mAb	Cell Signaling Technology	Cat#4661; RRID:AB_10557420
HSP60 (D307) Antibody	Cell Signaling Technology	Cat#4870; RRID:AB_2295614
ETFB Polyclonal antibody	Proteintech	Cat#17925-1-AP; RRID:AB_2100560
anti-α-tubulin antibody	Sigma-Aldrich	Cat#T5168; clone#B-5-1-2; RRID:AB_477579
anti-histone H3 antibody, CT, pan	Millipore	Cat#07-690; RRID:AB_417398
monoclonal anti-FLAG M2-Pertoxidase (HRP) antibody	Sigma-Aldrich	Cat#A8592; RRID:AB_439702
anti-HA antibody	Merck	Cat#11867423001; Clone#3F10; RRID:AB_390918
V5-tag Polyclonal antibody	Proteintech	Cat#14440-1-AP; RRID:AB_2878059
anti-FLAG M2 Affinity Gel	Sigma-Aldrich	Cat#A2220; RRID:AB_10063035

**Bacterial and virus strains**		

BL21(DE3)pLysS	Invitrogen	Cat#C606010

**Chemicals, peptides, and recombinant proteins**		

Doxycycline	Takara Bio	Cat#631311
Tet System Approved FBS	Clontech	Cat#631101

**Critical commercial assays**		

XFe96/XF Pro FluxPak	Agilent	Cat#103792-100
MitoSOX™ Green Mitochondrial Superoxide Indicator	Invitrogen	Cat#M36005
Mitotracker Green FM	Invitrogen	Cat#M7514

**Deposited data**		

Mitochondrial acetylome for eMAT substrates	This paper	PXD061245
mitochondria SILAC-LC-MS/MS for eMAT substrates	This paper	PXD061246

**Experimental models: Cell lines**		

HEK293T cells	ATCC	CRL-3216
hTERT-RPE1 cells	ATCC	CRL-4000

**Oligonucleotides**		

See Table S4 for oligonucleotides used in this paper	This paper	N/A

**Recombinant DNA**		

pCMVβ-p300-myc	a gift from Tso-Pang Yao	Addgene Plasmid #30489
pCMVβ-p300.DY-myc	a gift from Dr. Tso-Pang Yao	Addgene Plasmid #30490
pcDNA3-p300_HAT-FLAG	This paper	N/A
pcDNA3-p300_Core-FLAG	This paper	N/A
pcDNA3-P1_MTS-p300_HAT-FLAG (pcDNA3-eMAT-FLAG)	This paper	N/A
pcDNA3-P1_MTS-p300_Core-FLAG	This paper	N/A
pcDNA3-C4_MTS-p300_HAT-FLAG	This paper	N/A
pcDNA3-C4_MTS-p300_Core-FLAG	This paper	N/A
pcDNA3-S4_MTS-p300_HAT-FLAG	This paper	N/A
pcDNA3-S4_MTS-p300_Core-FLAG	This paper	N/A
pcDNA3-FS1_MTS-p300_HAT-FLAG	This paper	N/A
pcDNA3-FS1_MTS-p300_Core-FLAG	This paper	N/A
pcDNA3-CAMP-p300_HAT-FLAG	This paper	N/A
pcDNA3-CAMP-p300_Core-FLAG	This paper	N/A
PB-TRE-dCas9-KRAB-MeCP2	Andrea et al.^15^	Addgene Plasmid #122267
PB-TRE-empty	This paper	N/A
PB-TRE-neo-empty	This paper	N/A
PB-TRE-eMAT-FLAG	This paper	N/A
PB-TRE-neo-eMAT-FLAG	This paper	N/A
PB-TRE-eMAT-DY-FLAG	This paper	N/A
PB-TRE-eMAT-V5	This paper	N/A
pQCXIB	This paper	N/A
pcDNA3.1-SIRT3-myc	a gift from Dr. Akihiro Ito	N/A
pQCXIP-SIRT3-HA	This paper	N/A
pQCXIB-SIRT3-HA	This paper	N/A
pcDNA3-GCN5L1-FLAG	This paper	N/A
pcDNA3-KAT8-FLAG	This paper	N/A
pET19-His-ACO2-FLAG	This paper	N/A
pET19-His-NDUFS3-FLAG	This paper	N/A
pET19-His-eMAT-V5	This paper	N/A
pcDNA3-SDHA-FLAG	This paper	N/A
pcDNA3-OGDH-FLAG	This paper	N/A
pcDNA3-DLST-FLAG	This paper	N/A
pcDNA3-SUCLG1-FLAG	This paper	N/A
pcDNA3-SUCLA2-FLAG	This paper	N/A
pcDNA3-ACO2-FLAG	This paper	N/A
pcDNA3-DLAT-FLAG	This paper	N/A
pcDNA3-ATP5F1C-FLAG	This paper	N/A
pcDNA3-NDUFA6-FLAG	This paper	N/A
pcDNA3-MDH2-FLAG	This paper	N/A
pcDNA3-UQCRC1-FLAG	This paper	N/A
pcDNA3-NDUFS3-FLAG	This paper	N/A
pcDNA3-SOD2-FLAG	This paper	N/A
pL-CRISPR.EFS.GFP	Heckl et al.^50^	Addgene Plasmid #57818
pL-CRISPR.EFS.tRFP	Heckl et al.^50^	Addgene Plasmid #57819

**Software and algorithms**		

DAVID	Sherman et al.^52^	https://davidbioinformatics.nih.gov/
WebLogo	Crooks et al.^53^	https://weblogo.threeplusone.com/
ImageJ	Schneider et al.^58^	https://imagej.net/ij/
ColabFold	Mirdita et al.^14^	https://colab.research.google.com/github/sokrypton/ColabFold/blob/main/AlphaFold2.ipynb
Xcalibur Software	Thermo Fisher Scientific	https://www.thermofisher.com/order/catalog/product/OPTON-30965
Proteome Discoverer Software	Thermo Fisher Scientific	https://www.thermofisher.com/jp/ja/home/industrial/mass-spectrometry/liquid-chromatography-mass-spectrometry-lc-ms/lc-ms-software/multi-omics-data-analysis/proteome-discoverer-software.html?erpType=Global_E1
MASCOT search engine software	Matrix Science	https://www.matrixscience.com/
Excel	Microsoft	https://www.microsoft.com/en-ca/microsoft-365/excel
R software	R Core Team (2023)	https://www.R-project.org/

### Experimental model and study participant details

#### Cell lines

HEK293T (ATCC, #CRL-3216) and hTERT-RPE1 (ATCC, #CRL-4000) cells were cultured in Dulbecco’s Modified Eagle Medium (DMEM, Nacalai Tesque) or DMEM/Ham’s F-12 (Nacalai Tesque) supplemented with 10% fetal bovine serum (Japan Bioserum) and 100 U/mL penicillin-streptomycin solution (Thermo Fisher Scientific), and maintained under 5% CO2 at 37°C. For experiments involving doxycycline induction, eMAT-inducible cells were cultured in medium supplemented with 10% Tet System Approved FBS (Clontech, #631101) instead of the standard fetal bovine serum.

The absence of Mycoplasma contamination was routinely monitored using the MycoAlert Mycoplasma Detection Kit (Lonza Bioscience). The cell lines used in this study were obtained from ATCC and maintained under standard culture conditions; however, they have not been independently authenticated.

### Method details

#### Reagents and antibodies

All reagents used in this experiment were purchased from SIGMA or Nacalai Tesque unless otherwise stated. Antibodies used for lysine modification were; Anti-Succinyllysine rabbit pAb (PTM BIO, #PTM-401), Malonyl-Lysine multiMab Rabbit mAB mix (Cell Signaling, #14942), Acetylated Lysine (Ac-K2-100) MultiMab Rabbit mAb mix (Cell Signaling, #9814) and Acetylated-Lysine Antibody (Cell Signaling, #9441). SIRT3 (D22A3) Rabbit mAb (Cell signaling, #5490), COXIV mouse mAb (Cell Signaling clone#4D11-B3-E8, #11967), VDAC (D73D12) Rabbit mAb (Cell Signaling, #4661), HSP60 (D307) Antibody (Cell Signaling, #4870), ETFB Polyclonal antibody (Proteintech, #17925-1-AP), anti-α-tubulin antibody (clone B-5-1-2, Sigma), monoclonal anti-FLAG M2-Pertoxidase (HRP) antibody (Merck, #A8592), anti-HA antibody (clone 3F10, Roche), V5-tag Polyclonal antibody (Proteintech, #14440-1-AP). anti-FLAG M2 Affinity Gel (Merck, #A2220) was used for immunoprecipitation.

#### Plasmids

Plasmids for eMAT were constructed from human p300 cDNA fragments amplified from pCMVβ-p300-myc (addgene#30489), or pCMVβ-p300.DY-myc (addgene#30490) for the catalytic mutant. The amplified catalytic cDNA fragment (p300_HAT) with 5′-AgeI site was cloned into EcoRI and NotI sites of pcDNA3 vector (Invitrogen) with c-terminal FLAG tag or V5 tag sequence. Next, annealed oligonucleotides corresponding to MTS of various mitochondrial proteins were inserted into the EcoRI and AgeI sites of the obtained pcDNA3-p300_HAT plasmids. The MTS and Core domain fusion constructs were obtained by the replacement of the p300_HAT to the Core domain using AgeI and NotI sites. For Dox inducible expression, PB-TRE-dCas9-KRAB-MeCP2 (addgene#122267) was digested with NheI and PmeI, then inserted with a stuffer sequence containing multiple cloning sites to obtain PB-TRE-NheI-PacI-SnaBI-AgeI-PmeI vector (PB-TRE-empty). In-fusion cloning was performed to obtain the PB-TRE-neo-empty vector whose hygromycin resistance cassette was replaced with a neomycin resistance cassette. PCR amplified fragments from eMAT with FLAG or V5 were cloned into NheI and PmeI sites of the PB-TRE-empty or PB-TRE-neo-empty vector to obtain the Dox inducible plasmids (PB-TRE-eMAT-FLAG and PB-TRE-eMAT-V5). Human SIRT3 (GenBank: NM_012239) was amplified from pcDNA3.1-SIRT3-myc vector (kindly provided from Dr. Ito), and cloned into pcDNA3 vector or pQCXIP vector (Clontech) with c-terminal HA tag sequence. In-fusion cloning was performed for the pQCXIB vector whose puromycin resistance cassette was substituted with a blasticidin (BSD) resistance cassette. Other plasmids for mitochondria proteins were amplified from HEK293T cDNA unless mentioned, and cloned into pcDNA3 vector with c-terminal FLAG sequence. All sequences were confirmed by DNA sequencing. All primers used in this study were listed in Table S4.

#### Transfection and generation of stable cell lines

For transient transfections, HEK293T and hTERT-RPE1 cells were transfected using PEI transfection reagent (Polysciences, Inc.) and ViaFect transfection reagent (Promega), respectively.

For PiggyBac-mediated stable transfection, PB-TRE-eMAT plasmids were co-transfected with the transposase expression plasmid pCAGPBase (Addgene #40972) using the appropriate transfection reagent. Twenty-four hours after transfection, selection antibiotics were added to the culture medium: 400 μg/mL hygromycin B for HEK293T cells and 800 μg/mL G418 for hTERT-RPE1 cells. Cells were selected for an additional two weeks in the presence of the drug. The surviving and proliferating population (referred to as eMAT cells) was used for subsequent experiments.

For retroviral infection, the pQCXIP-SIRT3-HA plasmid (for HEK293T cells) or pQCXIB-SIRT3-HA plasmid (for hTERT-RPE1 cells), along with the corresponding empty vector, was co-transfected into HEK293T cells together with gag-pol-env expression constructs. Twenty-four hours after transfection, virus-containing culture supernatants were collected, filtered through a 0.45 μm syringe filter, and transferred to target eMAT cells in the presence of 4–8 μg/mL polybrene. After 24–48 hours, cells were selected with 1 μg/mL puromycin (HEK293T) or 10 μg/mL blasticidin S (BSD, hTERT-RPE1) for two weeks to generate SIRT3-HA–overexpressing eMAT cells (eMAT+SIRT3) and corresponding control cells (eMAT+vector).

Selection antibiotics were maintained in the culture medium every 2–3 passages throughout the experiments.

#### Generation of *SIRT3* KO cells

*SIRT3* KO HEK293T cells were generated with a CRISPR-Cas9-mediated gene editing. Two guide RNAs targeting exon 4 (5′-ATGAGCTTCAACCAGCTTTG-3′ and 5′-CTCACCCGAATGTCCTCCCC-3′) were cloned into pL-CRISPR.EFS.GFP vector (Addgene#57818) and pL-CRISPR.EFS.tRFP vector (Addgene#57819),^50^ respectively. The plasmids (pL.CRISPR.GFP-gSIRT3 and pL.CRISPR.tRFP-gSIRT3) were transfected into HEK293T cells using PEI transfection reagent, and GFP/RFP double-positive cells were sorted and plated onto 96-well plates. The KO clones were screened by genomic PCR. All primers used in this study are listed in Table S4.

#### Mitochondria and sub-mitochondria fractionation

Mitochondria fractionation was performed as described previously.^8^ In brief, HEK293T cells or hTERT-RPE1 cells were cultured in a 150-mm dish (containing approx. 1.5-3×10ˆ7 cells), washed with 10 mL 1×PBS, and harvested with a cell scraper in 1 mL ice-cold 1×PBS. The cells were centrifuged at 400 g for 3 min, and the cell pellets were resuspended in 1 mL of SEM buffer (50 mM Sucrose, 1 mM EDTA, 200 mM Mannitol, 10 mM HEPES-KOH, pH 7.5) with protease inhibitor cocktail for homogenization. The resuspended cells were transferred to a Dounce homogenizer (KONTES) and homogenized at 20 strokes with pestle A on ice. The homogenized lysates were centrifuged at 700 g for 10 min to precipitate debris and nuclei, and the sup was collected in a new tube. The sup was centrifuged at 700 g for 10 min again, and the sup was transferred to another new tube, then centrifuged at 7,000 g for 10 min to precipitate mitochondria-enriched fractions. The mitochondrial pellet (ppt) was washed once with 1 mL of SEM buffer, and the ppt was stored in -80°C freezer for downstream experiments, or used immediately for sub-mitochondria fractionation.

Submitochondrial fractionation was performed as described^8^^,^^51^ with some modifications. For carbohydrate extraction of membrane proteins, 100 mM sodium carbonate buffer (pH 11.5) was added to isolated mitochondria, and incubated for 20 min on ice. The lysates were ultra-centrifuged at 124,000 g (55,000 rpm) for 30 min at 4°C (Beckman, TLA-100.3). The sup was collected in a new tube, precipitated with 10% TCA, and solubilized with 1×SDS sample buffer, and the pellet was directly lysed with 1×SDS sample buffer for immunoblotting.

For the mitoplast experiment, a hypotonic buffer (20 mM HEPES-KOH) was added to isolated mitochondria and incubated on ice for 15 min to burst the outer membrane. The mitoplast and untreated mitochondria in SEM buffer were incubated with 100 ng/mL of proteinase K for 10 min on ice to remove membrane surface-bound proteins. The PK reaction was stopped by adding a 1×protease inhibitor cocktail, and the mitoplast and mitochondria were pelleted by centrifugation (10,000 g for 10 min), lysed with 1×SDS buffer, and analyzed by immunoblotting.

#### Immunofluorescence microscopy

eMAT-inducible cells were cultured with the indicated amount of Dox (0-100 ng/mL) on a coverslip for 24 h. The cells were washed once with 1×PBS and fixed with 3.7% HCHO/1×PBS for 10 min at 25°C. Fixed cells were permeabilized with 0.5% TritonX-100/1×PBS for 10 min and blocked with 5% bovine serum albumin in 1×PBS for 30 min at 25°C. Fixed cells were incubated with primary antibodies for 1 h, and stained with Alexa Fluor-conjugated secondary antibodies (Alexa Fluor® Goat Anti-Mouse IgG (H+L) Antibody, Life Technologies). For the mitochondrial marker, anti-ETFB antibody was used to visualize the mitochondrial outer-membrane protein ETFβ. Fluorescent images were obtained with a confocal microscopy FV-3000 (Olympus).

#### Cell cycle analysis

For cell cycle analysis, 5×10^5^ eMAT-inducible cells in a 6 cm dish were treated with or without 10 ng/mL Dox for 48 h. Cells were collected and fixed with 500 μL of 70% EtOH, then 500 μL of 10 μg/mL RNaseA in 1×PBS were added and incubated at 37°C for 15 min. Cells were centrifuged and the pellets were tapped gently to prevent the cells from clumping together, and stained with 10 μg/mL propidium iodide (PI) in 1×PBS. Cell cycle analysis was performed with a flow cytometer (BD, FACSymphony™ A3).

#### Live cell counting with a flow cytometer

eMAT-inducible cells were treated with or without 10 ng/mL Dox for 48 h. Floating cells in the culture medium and attached cells were collected in a single tube and stained with 1 μg/mL PI. PI-positive dead cells and PI-negative live cells were quantified using a flow cytometer (BD, FACSymphony A3).

#### Mitochondrial acetylome

Each isolated mitochondria (n=1) from HEK293T cells were dissolved in 100 mM ammonium bicarbonate (ABC) buffer and sonicated twice for 15 sec each. 250 μg of sonicated mitochondrial protein lysates were incubated with 30 mM DTT for 30 min at 56°C, followed by incubation with 30 mM iodoacetamide for 30 min at 37°C in the dark. The lysates were digested with 5 μg of mass spectrometry grade trypsin (Trypsin Gold, Promega) for 24 h at 37°C under gentle rotation with a thermomixer (1000 rpm). The trypsin-digested peptides were concentrated and dried up with a Speed-Vac, and resolved in 1mL of IP buffer (50 mM Tris-HCl with pH 8.0, 400 mM NaCl, 0.5% Nonidet P-40 with Protease inhibitor cocktail (Roche)), and centrifuged for 16,000 g for 10 min to precipitate undigested debris. The sups were incubated with a cocktail of antibodies containing 2.5 μL of Acetylated Lysine (Ac-K2-100) MultiMab Rabbit mAb mix (Cell Signaling, #9814) and 2.5 μL of Acetylated-Lysine Antibody (Cell Signaling, #9441) for one hour with gentle agitation. 20 μL of protein A/G agarose beads (Santacruz, sc-2003) were added and incubated for another hour. The peptide-bound beads were washed three times with IP buffer, then washed twice with 100 mM ABC buffer, and transferred to a new tube. The peptides were first eluted with 0.1% TFA and then eluted again with 0.1% TFA/75% acetonitrile. The eluate was concentrated and dried up with Speed-vac, filtered with ZIP-tip SCX, and the peptide fragments were applied to a liquid chromatograph (Vanquish Neo; Thermo Fisher Scientific, Odense, Denmark) coupled to an Orbitrap Eclipse Mass Spectrometer (Thermo Fisher Scientific, Inc., San Jose, CA, USA), with a nanospray ion source in positive mode. The peptides were separated on a NANO-HPLC C18 capillary column (0.075-mm inner diameter × 150 mm length, 3 mm particle size; Nikkyo Technos, Tokyo, Japan). Mobile phase “A” was comprised of water with 0.1% formic acid, and mobile phase “B” was comprised of acetonitrile with 0.1% formic acid. Two different slopes were used for a gradient elution for 120 min at a flow rate of 300 nL/min: 0%–30% of phase B in 100 min and 30%–65% of phase B in 20 min. The mass spectrometer was operated in the FAIMS method with 1 s cycle time per CV for a total of 3 s cycle time (-40, -60, -80 V). The parameters for operating the mass spectrometer were as follows: spray voltage, 1.9 kV; capillary temperature, 275°C; mass-to-charge ratio, 350-1800; normalized collision energy, 30%. Raw data was acquired using the Xcalibur software (Thermo Fisher Scientific). The MS and MS/MS data were searched against the Swiss-Prot database using Proteome Discoverer 3.1 (Thermo Fisher Scientific) using the MASCOT search engine software version no. 2.8.0 (Matrix Science, London, United Kingdom). Peptides containing at least one acetylated lysine with false discovery rates (FDR) of less than 5% were defined as hit peptides (Table S1). A non-label quantification was performed according to the manufacturer’s standard protocol (Proteome Discoverer version 3.1, Thermo Fisher Scientific). GO analysis and statistical analysis were performed with DAVID^52^ (Ver.2021) and R software.

#### Immunoblot analysis and LC-MS/MS of acetylated proteins

The plasmids for C-terminally FLAG-tagged proteins (pcDNA3-X-FLAG) were transfected into eMAT-V5+vec or eMAT-V5+SIRT3 cells. 24 hours after the transfection, the cells were treated with or without 10 ng/mL of Dox and cultured for 24 h. The cells were harvested and lysed in IP buffer (50 mM Tris-HCl pH7.5, 400 mM NaCl, 0.5% NP-40, and protease inhibitors). M2-agarose beads (Sigma-Aldrich) were added and incubated with the lysate for 1 hour at 4°C, then washed three times with IP buffer. For immunoblot analysis, the bound proteins were separated with SDS-PAGE and transferred to a PVDF membrane, and their acetylation was detected with immunoblot with anti-AcK antibodies (Cell Signaling, #9814). For LC-MS/MS, the gel band corresponding to FLAG-tagged proteins was excised and in-gel digested with trypsin unless described. The trypsinized protein fragments were applied to a liquid chromatograph (EASY-nLC 1200) coupled to a Q Exactive HF-X Hybrid Quadrupole-Orbitrap Mass Spectrometer. The acquired data was processed using MASCOT 2.8 and Proteome Discoverer 3.1. The search parameters for MS and MS/MS data were as follows: enzyme, trypsin; static modifications, none; dynamic modifications, oxidation (Met), Acetylation (Lys), propionamide (Cys) Acetyl (Protein N-term), Gln>; precursor mass tolerance, ± 15 ppm; fragment mass tolerance, ± 30 mmu; maximum missed cleavages, 3, Instrument type: ESI-TRAP. The proteins were considered identified when their false discovery rates (FDR) were less than 1%. Normalized acetylation (%) was calculated based on the intensity of the acetylated peptide, normalized by the sum of the intensities of the corresponding unacetylated and acetylated peptides. Consensus motif analysis was performed with WebLogo^53^ (Ver.3).

#### Metabolome analysis

For TCA metabolites, approximately 2×10ˆ6 cells in a 6-well plate were washed once with ice-cold 1×PBS, then 500 μL of ice-cold 70% MeOH per 2×10ˆ6 cells with 1 μM internal standard mix (13C_6_-G6P, d4-Succinate, 13C_6_-Glc) was directly added into the culture plate to stop metabolic reaction immediately. The volume of 70% MeOH added was corrected according to the cell number cultured on a separately prepared culture plate. The 70% MeOH treated cells were harvested with a cell scraper, sonicated for 1 min, and then centrifuged at 16,000 g for 10 min. The MeOH-extracted sup was analyzed with MRM LC-MS/MS (Thermo Fisher Scientific, TSQ Altis). Acetyl-CoA measurements were performed according to the established protocol.^54^ Briefly, approximately 2×10ˆ6 cells in another 6 well plate cultured at the same time as the TCA metabolite experiment were washed once with ice-cold 1×PBS, and harvested with a cell scraper. The cell pellets were immediately lysed with 2.5% 5-Sulfosalicylic acid (SSA) with 1 μM internal standard mix (125 μL per 2×10^6^ cells), sonicated for 1 min, and centrifuged at 16,000 g for 10 min to extract CoA metabolites. The sup was analyzed with MRM LC-MS/MS (Thermo Fisher Scientific, TSQ Altis). Each metabolite was normalized with internal standard and quantified using external calibration curves, and obtained as pmol/L×10ˆ6 cells. n=3, mean±SEM. Statistical analysis was performed with the Tukey-HSD test. For acetyl-CoA measurement (Figure S6B), protein concentration was measured with a Bradford assay, and calculations of pmol/mg protein to molar concentrations were done assuming protein concentrations to be 200 mg/mL in cells.

#### Measurement of mitochondrial respiration

Mitochondrial respiration analysis was performed with an XFe96 analyzer (Agilent). HEK293T cells (2×10^4^ cells/well in 80 μL DMEM with the indicated amount of Dox) seeded in microplates were incubated overnight at 37 °C under 5% CO_2_. The culture medium was replaced with assay medium (seahorse XF DMEM Medium, pH 7.4 (Agilent) supplemented with 10 mM glucose (Agilent), 1 mM pyruvate (Agilent), and 2 mM Glutamine (Agilent)), after which the cells were incubated for 60 min at 37 °C in a CO_2_-free incubator. Three baseline measurements of the oxygen consumption rate (OCR) and extracellular acidification rate (ECAR) were taken using the Seahorse XFe96 analyzer, after which 25 μL of assay medium containing Oligomycin (2 μM) was injected from port A to start the Cell Mito Stress Test. This was followed by sequential treatments with carbonyl cyanide-p-trifluoromethoxyphenylhydrazone (FCCP, 1 μM) from port B and Rotenone/antimycin A (0.5 μM) from port C. Each assay cycle consisted of 3 minutes of mixing, 3 minutes of incubation, and 3 minutes of OCR measurements. For each condition, at least three replications were used to determine the average OCR. Three independent experiments were performed.

#### COXI and COXII activity assay

COXI^55^ and COXII^56^ activity assays were performed using established methods with minor modifications. Briefly, COXI assay was performed with 5-10 μg of mitochondria lysates sonicated in 1×PBS supplemented with 2 mg/mL dodecyl β-d-maltoside by mixing in a reaction buffer containing 25 mM potassium phosphate, pH 7.5, 3.5 mg/mL BSA, 75 μM DCPIP, 80 μM decylubiquinone, 40 mM NaN_3_, and 0.2 mM NADH. For COXII assay, 5-10 μg of mitochondria lysates were mixed with a reaction buffer containing 25 mM potassium phosphate pH 7.5, 1 mg/mL BSA, 75 μM DCPIP, 40 μM decylubiquinone, 40 mM NaN_3_, and 20 mM sodium succinate. The enzymatic activity of COXI/COXII was assessed by monitoring the reduction of DCPIP at 600 nm in 30-second intervals over 10 minutes at 37°C using a spectrophotometer (Molecular Devices, SpectraMax 190). The recorded activity was normalized to the protein amount, as determined by the Bradford Protein Assay Kit (BioRad Laboratories, Hercules, CA, USA).

#### Mitochondrial DNA copy number

Mitochondrial DNA copy number were determined as described.^57^ Briefly, five ng of isolated total DNA (n=3) were mixed with 400 nM target primer mix and 7.5 μL of Power SYBR Green PCR Master Mix (Thermo Fisher scientific) in 15 μL reaction volume, and detected with a StepOne realtime PCR System (Applied Biosystems). The primer sequences for mtDNA (mitochondrial tRNALeu^(UUR)^) and nucDNA (nuclear β2M) are listed in Table S4. The obtained C_T_ values were used to determine relative mtDNA copy number to nucDNA with the following equation;

$$\Delta C_{T}=\left(\text{nucDNA}\;C_{T}\;–\;\text{mtDNA}\;C_{T}\right)$$

Relative mitochondria DNA copy number = 2 × 2ˆΔC_T_

#### Mitochondrial ROS measurement

Mitochondrial ROS was measured with a MitoSOX™ Green Mitochondrial Superoxide Indicator (Invitrogen, M36005). Control, eMAT or eMAT+SIRT3 cells were cultured in the presence of 100 ng/mL Dox for 7 days, and seeded on a 35 mm Glass Bottom Dish (MATTEK) the day before assay. Cells were washed with 1xHBSS, then incubated with MitoSOX Green (10 uM) in 1xHBSS for 30 min at 37°C and 5% CO.^2^ The stained cells were washed 3 times with 1xHBSS, and their fluorescence was observed under a microscope (Olympus, FV 3000).

Obtained fluorescent images were quantified using ImageJ software.^58^ At least 200 cells were quantified in each experiment (n=3).

#### SA-βGal staining

Senescence-associated β-galactosidase (SA-βGal) was visualized according to the established method.^29^ In brief, the eMAT-inducible hTERT-RPE1 cells were cultured in the presence or absence of dox (100 ng/mL) for 7 days, then cells in a 6-well plate were washed twice with 1×PBS and fixed with 3.7% HCHO/1×PBS for 10 min at 25°C. The fixed cells were stained with Staining buffer (40 mM citric acid/Na phosphate buffer, 5 mM K_4_[Fe(CN)_6_] 3H_2_O, 5 mM K_3_[Fe(CN)_6_], 150 mM sodium chloride, 2 mM magnesium chloride and 1 mg/mL X-gal) for 16 h at 37°C. Cells were washed twice with 1×PBS, and observed under a microscope with bright field. For SA-βGal positive cell counting, at least 100 cells were counted per experiment. n=6 for each experimental condition.

#### RT-qPCR

RNAs were extracted from hTERT-RPE1 cells using Sepasol-RNA I Super G (Nacalai Tesque). Reverse transcription was performed using Omniscript RT Kit (QIAGEN) according to the manufacturer’s instructions. For quantitative PCR, cDNA fragments were amplified with Power SYBR Green Master Mix (Thermo Fisher scientific) and detected with a StepOne realtime PCR System (Applied Biosystems). Relative expression was calculated using the 2−ΔΔCt method. GAPDH was used as a reference gene. The primer sequences are listed in Table S4.

#### Acid extraction of histones

Histones were extracted with acid extraction methods. Briefly, 1x10^6^ cell pellets were incubated with 100 μL of 0.2N HCL at 1,000 rpm overnight at 4°C. The lysates were centrifuged at 2000 rpm for 10 min, and the sup was collected in a new tube, quantified with the Bradford assay, then neutralized with 1/5 vol. of 2 M Tris-base. The acetylation of histones was analyzed with immunoblotting with anti-AcK antibodies (Cell Signaling, #9814) and anti-histone H3 antibody, CT, pan (Cell Signaling, #07-690) as loading control.

#### SILAC-LC-MS/MS analysis for acetylated proteins

Control cells with PB-TRE-empty vector or eMAT cells were cultured in either light Arg and Lys or heavy isotope-labeled Arg (^13^C_6_^15^N_4_ L-Arginine) and Lys (^13^C_6_^15^N_2_ L-Lysine) for at least six doubling times (Thermo Fisher Scientific, SILAC Protein Quantitation Kit (Trypsin), DMEM A33972). Cells were then treated with 10 ng/mL Dox for 48 h, after which mitochondria were isolated (n=3). The mitochondrial fractions were lysed in IP buffer containing 1× PBS supplemented with 0.5% NP-40, 1× protease inhibitor cocktail, and 5 mM sodium butyrate, followed by sonication for 10 sec. After centrifugation to remove debris, equal amounts (250 μg) of the supernatant from light-labeled mitochondria (control) and heavy-labeled mitochondria (eMAT) were mixed. The mixed lysates were incubated with a cocktail of antibodies containing 5 μL of Acetylated Lysine (Ac-K2-100) MultiMab Rabbit mAb mix (Cell Signaling, #9814) and 5 μL of Acetylated-Lysine Antibody (Cell Signaling, #9441) for 1 h with gentle agitation. 50 μL of protein A/G agarose beads (Santacruz sc-2003) were added and incubated for another 1 h. The protein-bound beads were washed three times with IP buffer, then washed twice with 1× PBS, and transferred to a new tube. DTT (20 mM) was added to protein-bound beads in 100 mM ABC buffer, and the mixture was incubated for 30 min at 56°C. Then, iodoacetamide was added and the mixture was incubated for 30 min at 37°C in the dark. The protein samples were then digested with 1 μg trypsin (Promega). The peptide fragments were applied to a liquid chromatograph (Vanquish Neo; Thermo Fisher Scientific, Odense, Denmark) coupled to an Orbitrap Eclipse Mass Spectrometer (Thermo Fisher Scientific, Inc., San Jose, CA, USA), with a nanospray ion source in positive mode. The peptides derived from the protein fragments were separated on a NANO-HPLC C18 capillary column (0.075-mm inner diameter × 150 mm length, 3 mm particle size; Nikkyo Technos, Tokyo, Japan). Mobile phase “A” was comprised of water with 0.1% formic acid, and mobile phase “B” was comprised of acetonitrile with 0.1% formic acid. Two different slopes were used for a gradient elution for 120 min at a flow rate of 300 nL/min: 0%–30% of phase B in 100 min and 30%–65% of phase B in 20 min. The mass spectrometer was operated in the FAIMS method with 1 s cycle time per CV for a total of 3 s cycle time (-40, -60, -80 V). The parameters for operating the mass spectrometer were as follows: spray voltage, 1.9 kV; capillary temperature, 275°C; mass-to-charge ratio, 350-1800; normalized collision energy, 30%. Raw data were acquired using the Xcalibur software (Thermo Fisher Scientific). The MS and MS/MS data were searched against the Swiss-Prot database using Proteome Discoverer 3.1 (Thermo Fisher Scientific) using the MASCOT search engine software version no. 2.8.0 (Matrix Science, London, United Kingdom). The peptides were considered identified when their false discovery rates (FDR) were less than 5%. For substrate identification, proteins exhibiting at least a 2-fold enrichment were defined as positive hit proteins. Three biological replications were analyzed to obtain the abundance ratio ((Heavy)/(Light), Table S3).

#### *In vitro* acetylation of eMAT substrates

For bacterial expression of His-tagged proteins, full-length ACO2-FLAG, full-length NDUFS3-FLAG, and eMAT-V5 without PDHA1-MTS domain were amplified from corresponding pcDNA3 vectors, and the fragments were cloned into BamHI-NdeI site in pET19b vector using In-Fusion HD Cloning Kit (Clontech). All primers used in this study are listed in Table S4. BL21(pLysS) strain was transformed with the pET19 vectors, cultured in 2×YT medium with ampicillin (100 μg/mL) and 0.2 mM isopropyl β-D-1-thiogalactopyranoside (IPTG) for 18 h at 18°C. The cells were pelleted and lysed with 1× PBS/0.5% NP-40 by sonication with a Branson Sonifier (S-250D, Branson Ultrasonics Corp., CT, USA) for 5 min on ice. The lysates were centrifuged at 15,000 g for 10 min, and supernatants were incubated with Ni-NTA Agarose (Qiagen, Valencia, CA, USA) for 1 h at 4°C with gentle agitation. The agarose beads were washed 5 times with wash buffer (50 mM Tris-HCl, pH 7.4, 25 mM imidazole) and eluted with the elution buffer (50 mM Tris-HCl, pH 7.4, 250 mM imidazole). The purified proteins were dialyzed with dialysis buffer (50 mM Tris-HCl, pH 8.0, 100 mM NaCl, 0.2 mM DTT, 10% glycerol) using an Amicon Ultra-15 centrifugal filter unit (Millipore, 10 kDa MWCO) and their concentration was measured using the Bradford assay. One microgram of His- and FLAG- tagged recombinant proteins, with or without 0.5 μg of recombinant His-eMAT-V5, was incubated in HAT reaction buffer^11^ containing 25 mM Tris-HCl, pH 7.5, 100 mM NaCl, 0.1 mM EDTA, 1 mM DTT, and varying amounts of acetyl-CoA (Sigma) at 37°C for 1 hour. The reactions were terminated by adding 1× SDS loading buffer, and acetylation levels were determined with immunoblotting with anti-AcK antibodies.

### Quantification and statistical analysis

Mean values, SD values, SEM values, and Student’s t-test (two-tailed unpaired) were calculated using Excel software (Microsoft). One-way ANOVA, Spearman correlation test, Dunnett’s test, and Tukey-Kramer post-hoc test were performed in R. P-values less than 0.1 were considered indicative of a trend, while those less than 0.05 were considered statistically significant. n.s.; not significant, p†<0.1, p∗ <0.05, p∗∗ <0.01, p∗∗∗ <0.001.

## Supplemental information

Document S1. Figures S1–S7 and Data S1

Table S1. Excel file containing a list of acetylome-identified peptides containing at least one AcK, related to Figure 4

Table S2. Excel file containing a list of eMAT substrates determined from Table S1, related to Figure 4

Table S3. Excel file containing a list of SILAC-LC-MS/MS-identified eMAT substrates, related to Figure S4

Table S4. Oligonucleotide primers used in this paper, related to STAR Methods (A) Oligonucleotide primers used for DNA cloning. (B) Oligonucleotide primers used for RT-qPCR. (C) Oligonucleotide primers used for CRISPR/Cas9-mediated gene editing.
