# Supplementary material for: Mitochondrial hyper-acetylation induced by an engineered acetyltransferase promotes cellular senescence
Source: iScience. 2025 Jul 29;28(9):113233. doi: 10.1016/j.isci.2025.113233 (PMC12357098; doi:10.1016/j.isci.2025.113233)
Supplement: Document S1. Figures S1–S7 and Data S1 [file mmc1.pdf]

iScience, Volume *28*

Supplemental information

Mitochondrial hyper-acetylation induced by an engineered acetyltransferase promotes cellular senescence

Tadahiro Shimazu, Ayane Kataoka, Takehiro Suzuki, Naoshi Dohmae, and Yoichi Shinkai

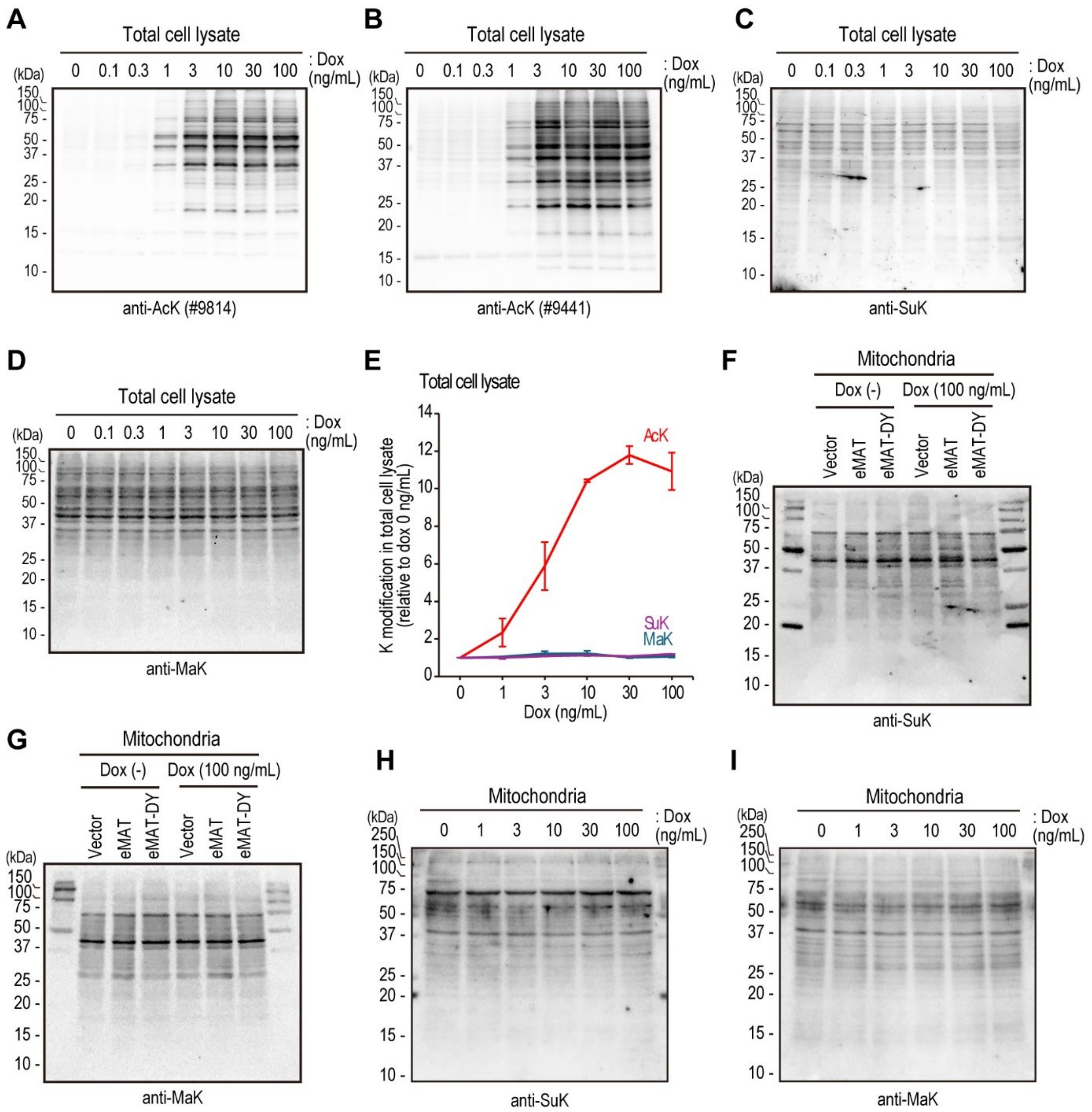

Figure S1. Lysine (K) acylations in eMAT cells, related to Figure 2.

(A-D) Dox-inducible eMAT cells were treated with the indicated concentrations of Dox for 24 h. K acylations in total cell lysate were assessed by immunoblotting using (A) anti-AcK antibodies (Cell Signaling, #9814), (B) anti-AcK antibodies (Cell Signaling, #9441), (C) anti-MaK antibodies (Cell Signaling, #14942), and (D) anti-SuK (PTM BIO, #PTM-401). Representative images are shown. The Dox-dependent eMAT expression in the total lysate and corresponding loading control are presented in Figure 2B. (E) Quantitation of K acylations in total cell lysate. Band intensities were quantified using

ImageJ software. n=2; mean±SEM. (F–I) Representative immunoblots of mitochondrial K acylations detected with (F,H) anti-SuK and (G,I) anti-MaK antibodies. Dox-dependent eMAT expression and the corresponding roading controls are shown in Figure 2E and Figure 2I, respectively.

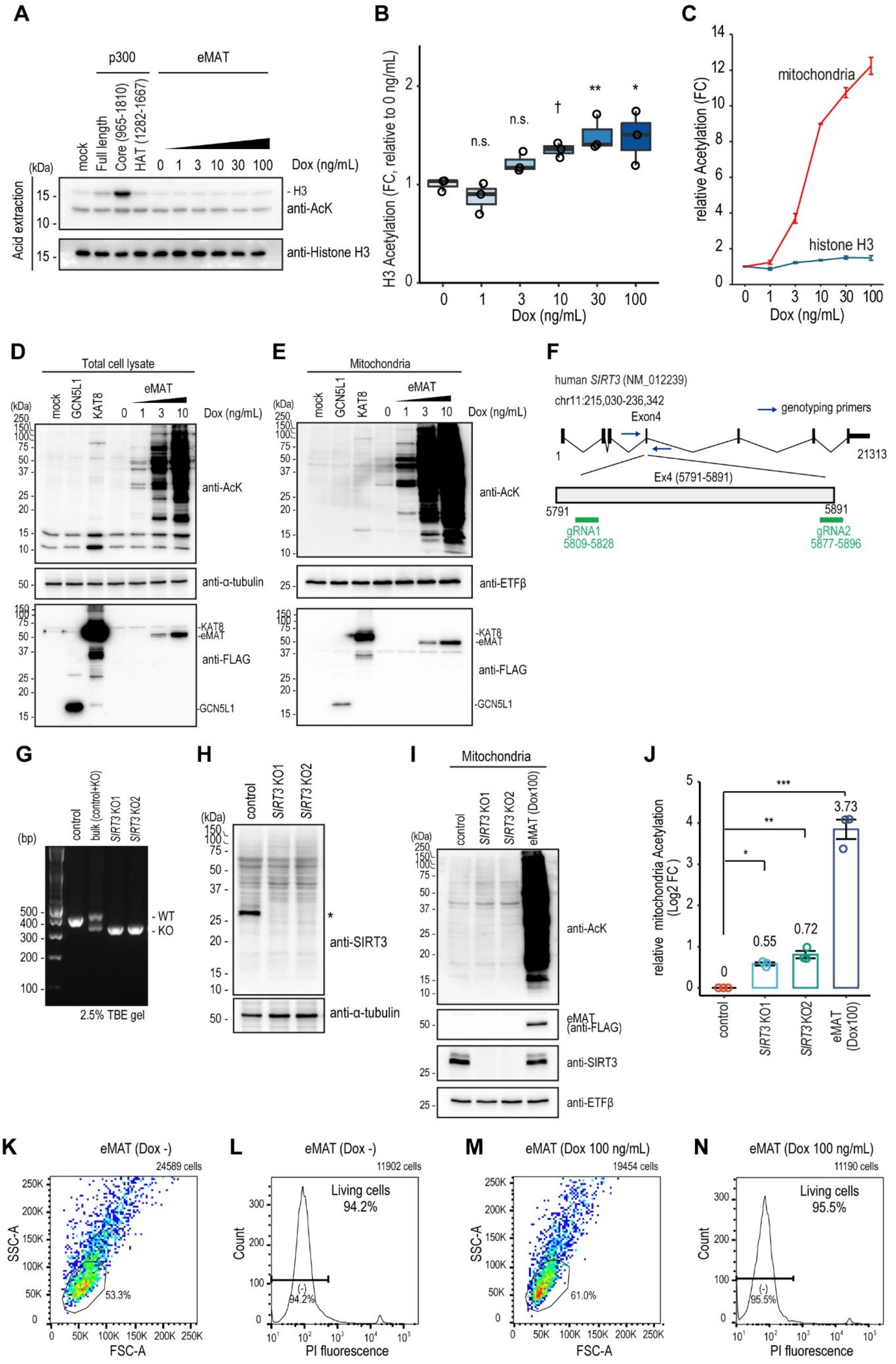
A
p300
eMAT
mock
Full length
Core (965-1810)
HAT (1282-1667)
0 1 3 10 30 100 Dox (ng/mL)
(kDa)
Acid extraction
- H3
anti-AcK
anti-Histone H3
B
H3 Acetylation (FC, relative to 0 ng/mL)
n.s.
n.s.
†
**
*
Dox (ng/mL)
C
relative Acetylation (FC)
mitochondria
histone H3
Dox (ng/mL)
D
Total cell lysate
mock
GCN5L1
KAT8
eMAT
Dox (ng/mL)
anti-AcK
anti-α-tubulin
-KAT8
-eMAT
anti-FLAG
-GCN5L1
E
Mitochondria
anti-ETFβ
F
human SIRT3 (NM_012239)
chr11:215,030-236,342
Exon4
genotyping primers
Ex4 (5791-5891)
gRNA1
5809-5828
gRNA2
5877-5896
G
(bp)
control
bulk (control+KO)
SIRT3 KO1
SIRT3 KO2
- WT
- KO
2.5% TBE gel
H
anti-SIRT3
I
eMAT (Dox100)
eMAT (anti-FLAG)
J
relative mitochondria Acetylation (Log2 FC)
0
0.55
0.72
3.73
K
eMAT (Dox -)
24589 cells
SSC-A
FSC-A
53.3%
L
11902 cells
Living cells
94.2%
Count
PI fluorescence
M
eMAT (Dox 100 ng/mL)
19454 cells
61.0%
N
11190 cells
Living cells
95.5%

Figure S2. eMAT is a potent engineered enzyme for mitochondrial acetylation, related to Figure 2. (A-C) Histone acetylation. HEK293T cells were transfected with indicated plasmids (p300 full length, Core domain, HAT domain) for 48 h. Dox-inducible eMAT cells were treated with the indicated concentrations of Dox for 24 h. (A) Histones were extracted from cells with the acid extraction method, and their acetylation was determined by immunoblotting with anti-AcK antibodies. (B) Quantitation of H3 acetylation. n=3; mean±SEM. Dunnett's test: p†<0.1, p*<0.05, p**<0.01. (C) Comparison of eMAT-induced mitochondrial acetylation (Figure 2J) and H3 acetylation (Figure S2B). (D-E) Comparison of the eMAT and other known mitochondria-associated acetyltransferases. HEK293T cells were transiently transfected with the plasmids for GCN5L1-FLAG or KAT8-FLAG and cultured for 48 h. The eMAT cells were treated with the indicated dose of Dox for 24 h. Acetylated proteins in the total cell lysate (D) or the mitochondria fraction (E) were determined with immunoblotting with anti-AcK antibodies. (F-J) Generation of *SIRT3* KO cells. (F) Schematic genomic sequence of *SIRT3* and the gRNA target sites. (G) Genotyping and (H) the immunoblot of WT and two independent *SIRT3* KO clones (KO1 and KO2). (I) A representative immunoblot image of anti-AcK antibodies in the mitochondria. (J) Quantitation of mitochondrial acetylation in the *SIRT3* KO clones and eMAT-expressing cells in the presence of Dox (100 ng/mL) for 24 h. mean±SEM; n=3. Dunnett's test: p*<0.05, p**<0.01, p***<0.001. (K-N) FACS analysis. (K, M) SSC vs FCS density plot and (L, N) histogram of PI fluorescence of unfixed eMAT cells treated with or without Dox (100 ng/mL) for 48 h.

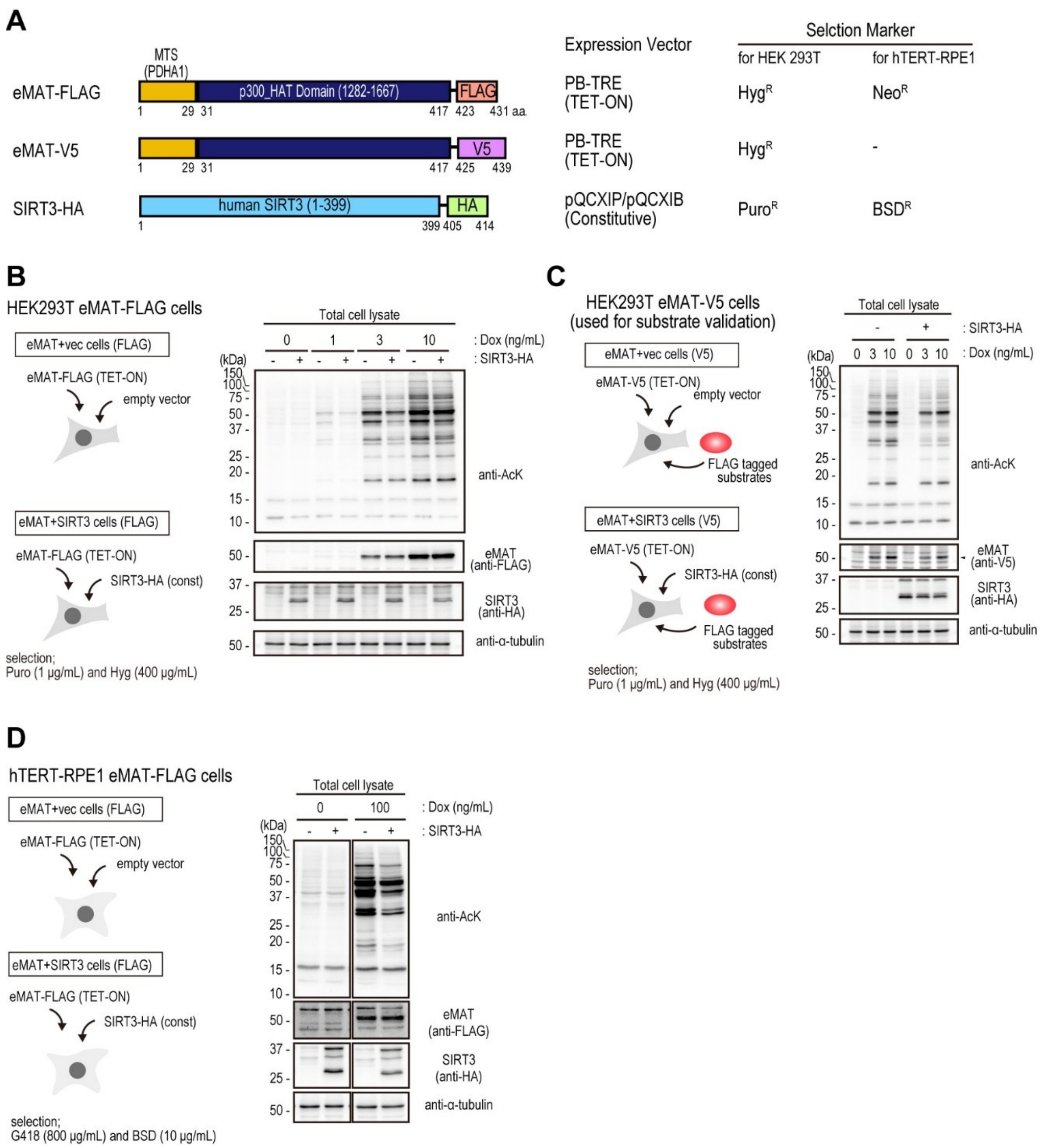

Figure S3. Dox-inducible eMAT cells, related to Figures 2-7.

(A) Schematic structure of eMAT constructs. (B-C) The indicated PB-TRE-eMAT-FLAG (B) or PB-TRE-eMAT-V5 (C) constructs were transfected into HEK293T cells with a piggyback-Dox inducible overexpression system. Clones were selected with hygromycin (400 µg/mL). Next, retrovirus containing SIRT3-HA or the empty vector (pQCXIP) was infected into the eMAT-HEK293T cells, and clones were selected with puromycin (1 µg/mL) and hygromycin for an additional 2 weeks to obtain

eMAT+SIRT3 cells. Acetylation level and the expression of eMAT and SIRT3 were confirmed by immunoblot. (D) PB-TRE-neo-eMAT-FLAG were transfected into hTERT-RPE1 cells, since hTERT-RPE1 cells are resistant to hygromycin and puromycin [S1]. Clones were selected with G418 (800 μg/mL) for 2 weeks. Retrovirus containing SIRT3-HA or the empty vector (pQCXIB) was infected into the eMAT-hTERT-RPE1 cells, and clones were selected with BSD (10 μg/mL) for 2 weeks to obtain eMAT+SIRT3 cells. Acetylation level and the expression of eMAT and SIRT3 were confirmed by immunoblot.

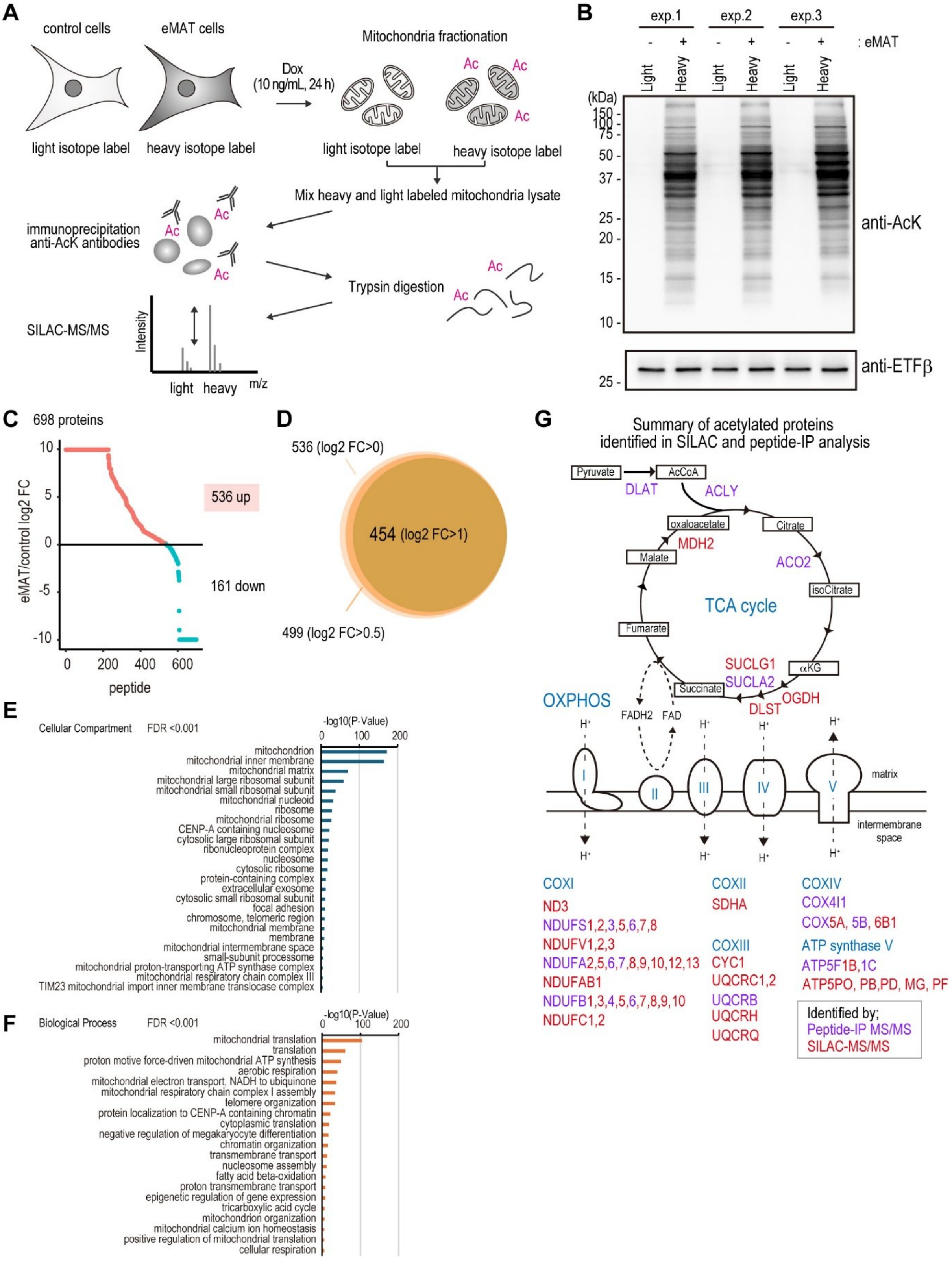
A
control cells
eMAT cells
light isotope label
heavy isotope label
Dox
(10 ng/mL, 24 h)
Mitochondria fractionation
Ac
light isotope label
heavy isotope label
Mix heavy and light labeled mitochondria lysate
immunoprecipitation
anti-AcK antibodies
Trypsin digestion
SILAC-MS/MS
Intensity
light
heavy
m/z
B
exp.1
exp.2
exp.3
: eMAT
Light
Heavy
(kDa)
150
100
75
50
37
25
20
15
10
anti-AcK
anti-ETFβ
C
698 proteins
eMAT/control log2 FC
536 up
161 down
peptide
D
536 (log2 FC>0)
454 (log2 FC>1)
499 (log2 FC>0.5)
E
Cellular Compartment FDR <0.001
-log10(P-Value)
mitochondrion
mitochondrial inner membrane
mitochondrial matrix
mitochondrial large ribosomal subunit
mitochondrial small ribosomal subunit
mitochondrial nucleoid
ribosome
mitochondrial ribosome
CENP-A containing nucleosome
cytosolic large ribosomal subunit
ribonucleoprotein complex
nucleosome
cytosolic ribosome
protein-containing complex
extracellular exosome
cytosolic small ribosomal subunit
focal adhesion
chromosome, telomeric region
mitochondrial membrane
membrane
mitochondrial intermembrane space
small-subunit processome
mitochondrial proton-transporting ATP synthase complex
mitochondrial respiratory chain complex III
TIM23 mitochondrial import inner membrane translocase complex
F
Biological Process FDR <0.001
-log10(P-Value)
mitochondrial translation
translation
proton motive force-driven mitochondrial ATP synthesis
aerobic respiration
mitochondrial electron transport, NADH to ubiquinone
mitochondrial respiratory chain complex I assembly
telomere organization
protein localization to CENP-A containing chromatin
cytoplasmic translation
negative regulation of megakaryocyte differentiation
chromatin organization
transmembrane transport
nucleosome assembly
fatty acid beta-oxidation
proton transmembrane transport
epigenetic regulation of gene expression
tricarboxylic acid cycle
mitochondrion organization
mitochondrial calcium ion homeostasis
positive regulation of mitochondrial translation
cellular respiration
G
Summary of acetylated proteins
identified in SILAC and peptide-IP analysis
Pyruvate
AcCoA
DLAT
ACLY
oxaloacetate
MDH2
Citrate
ACO2
Malate
isoCitrate
TCA cycle
Fumarate
αKG
SUCLG1
SUCLA2
Succinate
OGDH
DLST
OXPHOS
FADH2
FAD
matrix
intermembrane
space
COXI
ND3
NDUFS1,2,3,5,6,7,8
NDUFV1,2,3
NDUFA2,5,6,7,8,9,10,12,13
NDUFAB1
NDUFB1,3,4,5,6,7,8,9,10
NDUFC1,2
COXII
SDHA
COXIII
CYC1
UQCRC1,2
UQCRB
UQCRH
UQCRQ
COXIV
COX4I1
COX5A, 5B, 6B1
ATP synthase V
ATP5F1B,1C
ATP5PO, PB,PD, MG, PF
Identified by;
Peptide-IP MS/MS
SILAC-MS/MS

Figure S4. Acetylome analysis, related to Figures 4-5.

(A) A schematic for SILAC-MS/MS analysis of eMAT substrates. Light isotope-labeled control cells (PB-TRE vector) and heavy isotope-labeled eMAT-inducible cells were treated with 10 ng/mL Dox for 24 h. An equal amount of the light and heavy mitochondrial lysate was mixed in one tube. Acetylated proteins were immunoprecipitated with a cocktail of anti-AcK antibodies (#9441 and #9814) conjugated protein A/G agarose beads, and the beads-bound acetylated proteins were digested with trypsin on beads, then analyzed with LC-MS/MS. Three independent biological replicates were analyzed. (B) Aliquots of immunoprecipitated samples were analyzed with an immunoblot with anti-AcK antibodies. (C) Comparison of acetylation between eMAT and control cells. Fold change of acetylated proteins (total 698) were calculated as log2([protein abundance of eMAT]/[protein abundance of control]). 536 proteins were log2FC>0. (D) Ven diagram of the eMAT targets. Among 536 acetylated proteins with log2FC>0, 454 proteins with log2FC>1 were defined as eMAT substrates. GO analysis of eMAT targets with DAVID (Ver.2021): (E) Cellular compartment, (F) Biological process. (G) Summary of acetylated proteins identified in SILAC-MS/MS and peptide-IP MS/MS (Figure 4). See also Figure 4 and Tables S1-S3.

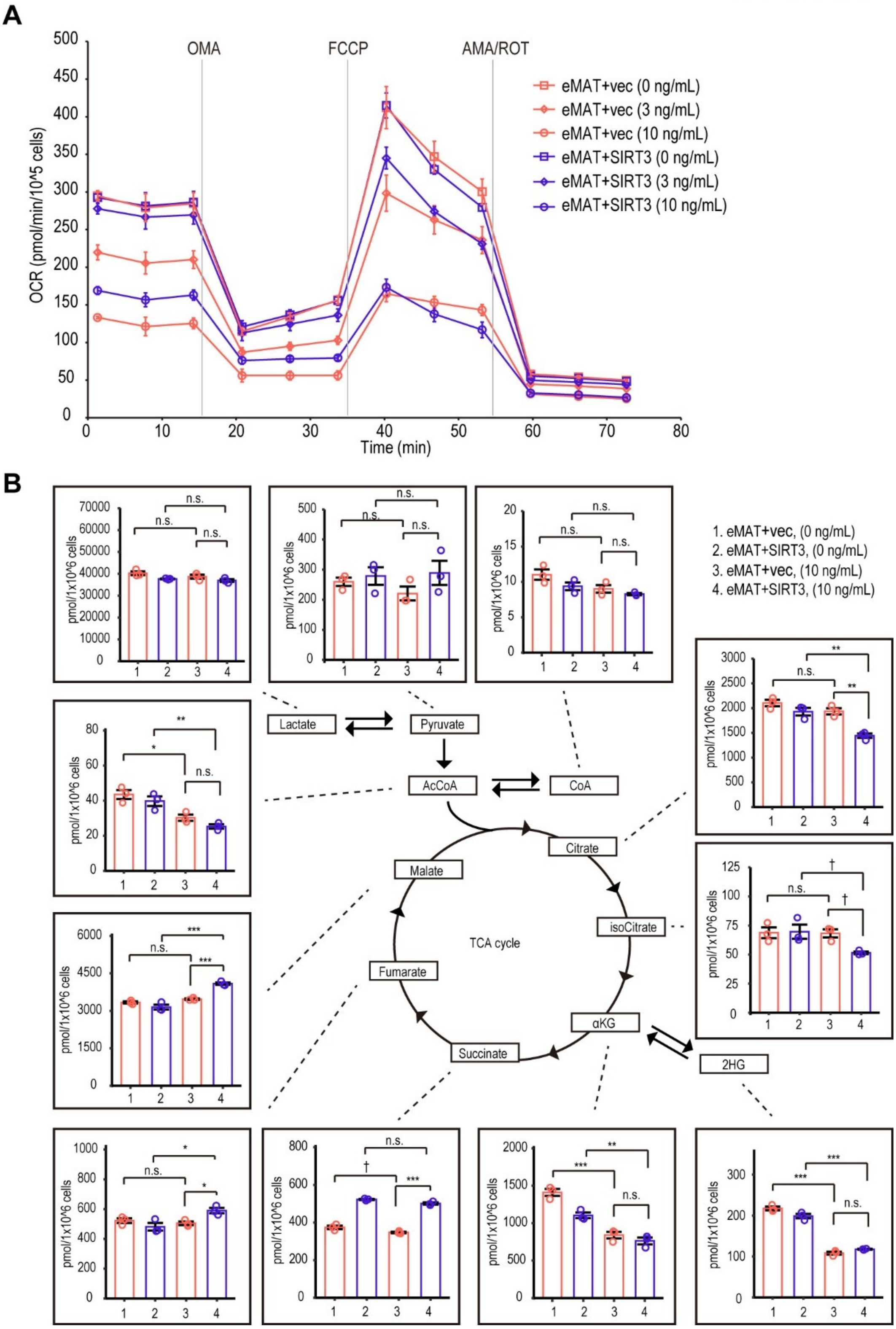
A
OMA
FCCP
AMA/ROT
OCR (pmol/min/10^5 cells)
Time (min)
eMAT+vec (0 ng/mL)
eMAT+vec (3 ng/mL)
eMAT+vec (10 ng/mL)
eMAT+SIRT3 (0 ng/mL)
eMAT+SIRT3 (3 ng/mL)
eMAT+SIRT3 (10 ng/mL)
B
1. eMAT+vec, (0 ng/mL)
2. eMAT+SIRT3, (0 ng/mL)
3. eMAT+vec, (10 ng/mL)
4. eMAT+SIRT3, (10 ng/mL)
pmol/1x10^6 cells
Lactate
Pyruvate
AcCoA
CoA
Citrate
isoCitrate
αKG
2HG
Succinate
Fumarate
Malate
TCA cycle
n.s.

Figure S5. eMAT regulates energy metabolism, related to Figure 6.

(A) Mitostress test was conducted with an extracellular flux analyzer. After three measurements of basal state, oligomycin A (OMA), FCCP, antimycin A (AMA) and rotenone (ROT) were injected into XFe96 assay plate to determine mitochondrial function. (B) Metabolome analysis. eMAT+vec or eMAT+SIRT3 cells were treated with 10 ng/mL Dox for 24 h, fixed, and the deproteinized supernatants were analyzed using MRM LC-MS/MS. n=3; mean±SEM. Tukey's HSD test: p†<0.1, p*<0.05, p**<0.01, p***<0.001. See also Figure 6.

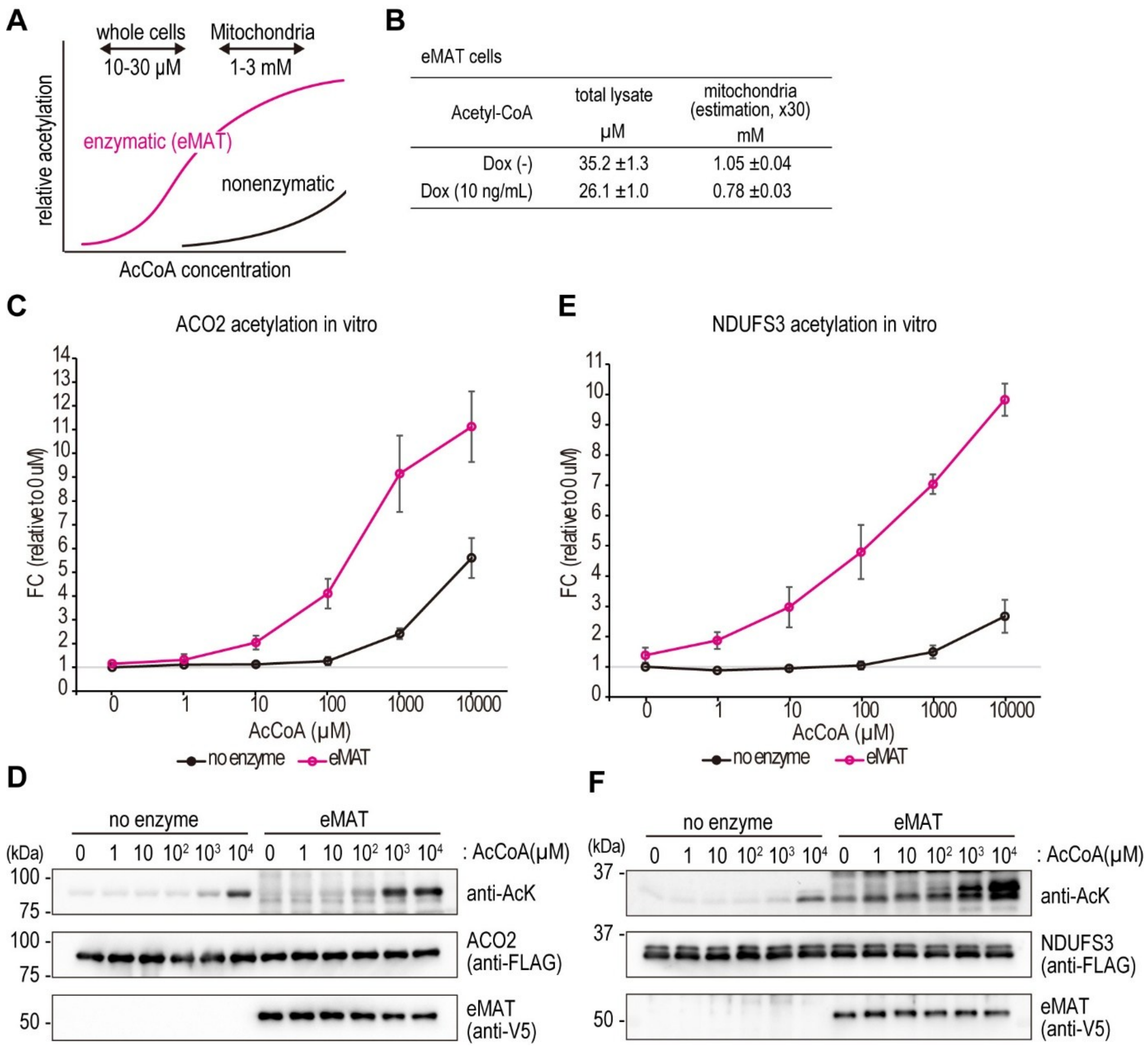

Acetyl-CoA	total lysate μM	mitochondria (estimation, x30) mM
Dox (-)	35.2 ±1.3	1.05 ±0.04
Dox (10 ng/mL)	26.1 ±1.0	0.78 ±0.03

Figure S6. eMAT can efficiently introduce acetylation, related to Figure 6.

(A) A schematic model of the dose-dependent enzymatic and nonenzymatic acetylation. (B) Acetyl-CoA concentration in eMAT (HEK293T) cells treated with or without 10 ng/mL Dox for 24 h. Mitochondrial concentration was estimated to be 30-fold that in total lysate, based on the previous report on yeast mitochondrial acetyl-CoA concentration [S2]. In vitro acetylation of (C-D) ACO2 and (E-F) NDUFS3. His-ACO2-FLAG or His-NDUFS3-FLAG were incubated with or without His-eMAT-V5 in the presence of indicated concentrations of acetyl-CoA (AcCoA), and incubated at 37°C for 2 h. Their acetylation was determined by immunoblotting with anti-AcK antibodies. Fold change of acetylation was calculated after normalization with the intensity of anti-FLAG using ImageJ software. n=3; mean±SEM.

A

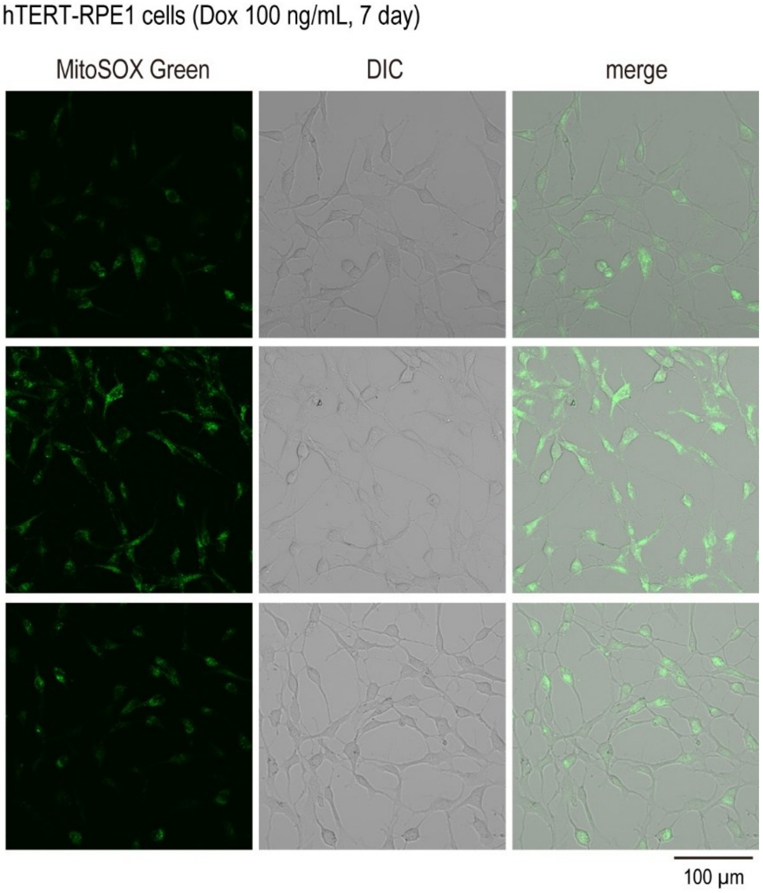
hTERT-RPE1 cells (Dox 100 ng/mL, 7 day)
MitoSOX Green
DIC
merge
control
eMAT
eMAT+
SIRT3
100 μm

B

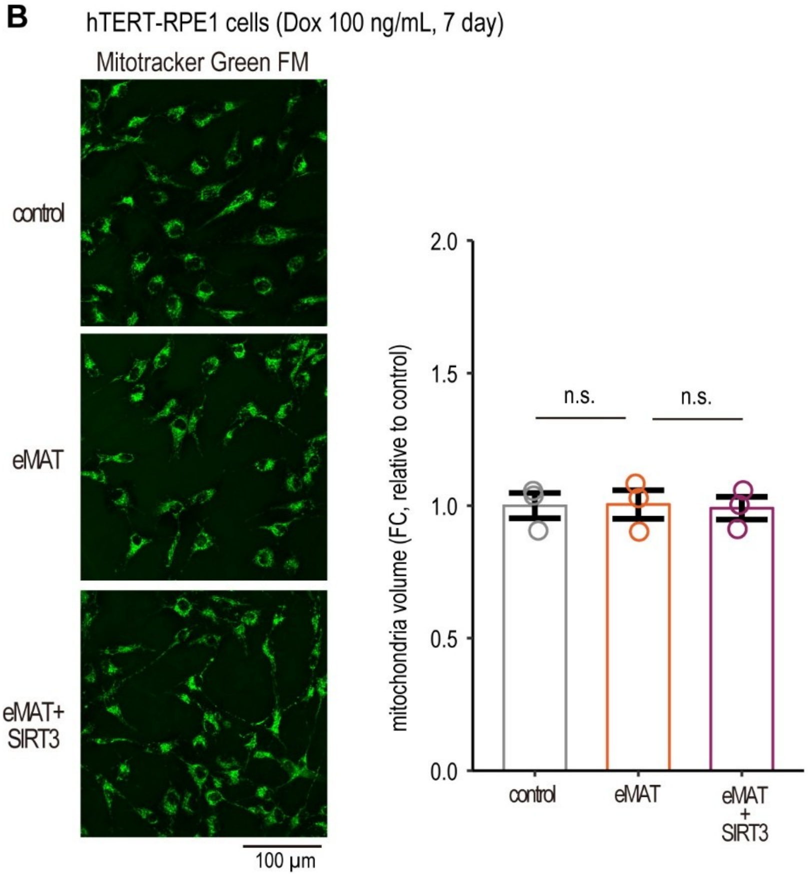
hTERT-RPE1 cells (Dox 100 ng/mL, 7 day)
Mitotracker Green FM
control
eMAT
eMAT+
SIRT3
100 μm
mitochondria volume (FC, relative to control)
2.0
1.5
1.0
0.5
0.0
n.s.
n.s.
control
eMAT
eMAT
+
SIRT3

Figure S7. Fluorescent images of hTERT-RPE1 cells stained with MitoSOX or Mitotracker Green FM, related to Figure 7.

(A) Control, eMAT and eMAT+SIRT3 hTERT-RPE1 cells were cultured with 100 ng/mL Dox for 7 days. Mitochondrial ROS were detected with a mitochondrial superoxide indicator (MitoSOX™ Green). Representative fluorescent images and DIC images were shown. Scale bar: 100 μm. See also Figure 7I. (B) Cells were stained with a mitochondria-specific fluorescent dye Mitotracker Green FM (Invitrogen, M7514), which is commonly used to assess mitochondrial mass[S3]. Scale bar: 100 μm. The fluorescent images were quantified with ImageJ software. At least 100 cells were counted in each experiment. n=3; mean±SEM. Tukey's HSD test: n.s.>0.1.

T.Shimazu et.al.

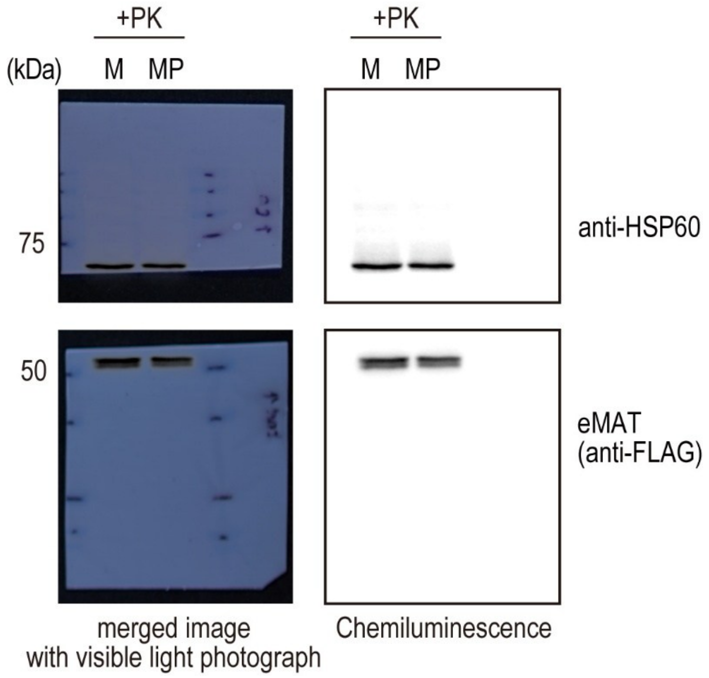

Data S1. Raw, uncropped images of the immunoblot membranes presented in Figure 2G.

Supplemental References

[S1]. Katoh, Y., Michisaka, S., Nozaki, S., Funabashi, T., Hirano, T., Takei, R., and Nakayama, K. (2017). Practical method for targeted disruption of cilia-related genes by using CRISPR/Cas9-mediated, homology-independent knock-in system. Mol Biol Cell *28*, 898-906. 10.1091/mbc.E17-01-0051.

[S2]. Weinert, B.T., Iesmantavicius, V., Moustafa, T., Scholz, C., Wagner, S.A., Magnes, C., Zechner, R., and Choudhary, C. (2014). Acetylation dynamics and stoichiometry in Saccharomyces cerevisiae. Mol Syst Biol *10*, 716. 10.1002/msb.134766.

[S3]. Wang, Q., Stringer, J.M., Liu, J., and Hutt, K.J. (2019). Evaluation of mitochondria in oocytes following gamma-irradiation. Sci Rep *9*, 19941. 10.1038/s41598-019-56423-w.
